# The Role of H1 Linker Histone Subtypes in Preserving the Fidelity of Elaboration of Mesendodermal and Neuroectodermal Lineages during Embryonic Development

**DOI:** 10.1371/journal.pone.0096858

**Published:** 2014-05-06

**Authors:** Giang D. Nguyen, Solen Gokhan, Aldrin E. Molero, Seung-Min Yang, Byung-Ju Kim, Arthur I. Skoultchi, Mark F. Mehler

**Affiliations:** 1 Roslyn and Leslie Goldstein Laboratory for Stem Cell Biology and Regenerative Medicine, Albert Einstein College of Medicine, Bronx, New York, United States of America; 2 Institute for Brain Disorders and Neural Regeneration, Albert Einstein College of Medicine, Bronx, New York, United States of America; 3 Department of Neurology, Albert Einstein College of Medicine, Bronx, New York, United States of America; 4 Department of Neuroscience, Albert Einstein College of Medicine, Bronx, New York, United States of America; 5 Department of Psychiatry and Behavioral Sciences, Albert Einstein College of Medicine, Bronx, New York, United States of America; 6 Rose F. Kennedy Center for Research on Intellectual and Developmental Disabilities, Albert Einstein College of Medicine, Bronx, New York, United States of America; 7 Einstein Cancer Center, Albert Einstein College of Medicine, Bronx, New York, United States of America; 8 Ruth L. and David S. Gottesman Institute for Stem Cell Biology and Regenerative Medicine, Albert Einstein College of Medicine, Bronx, New York, United States of America; 9 Center for Epigenomics, Albert Einstein College of Medicine, Bronx, New York, United States of America; 10 Institute for Aging Research, Albert Einstein College of Medicine, Bronx, New York, United States of America; 11 Department of Cell Biology, Albert Einstein College of Medicine, Bronx, New York, United States of America; Instituto de Medicina Molecular, Portugal

## Abstract

H1 linker histone proteins are essential for the structural and functional integrity of chromatin and for the fidelity of additional epigenetic modifications. Deletion of H1c, H1d and H1e in mice leads to embryonic lethality by mid-gestation with a broad spectrum of developmental alterations. To elucidate the cellular and molecular mechanisms underlying H1 linker histone developmental functions, we analyzed embryonic stem cells (ESCs) depleted of H1c, H1d and H1e subtypes (H1-KO ESCs) by utilizing established ESC differentiation paradigms. Our study revealed that although H1-KO ESCs continued to express core pluripotency genes and the embryonic stem cell markers, alkaline phosphatase and SSEA1, they exhibited enhanced cell death during embryoid body formation and during specification of mesendoderm and neuroectoderm. In addition, we demonstrated deregulation in the developmental programs of cardiomyocyte, hepatic and pancreatic lineage elaboration. Moreover, ectopic neurogenesis and cardiomyogenesis occurred during endoderm-derived pancreatic but not hepatic differentiation. Furthermore, neural differentiation paradigms revealed selective impairments in the specification and maturation of glutamatergic and dopaminergic neurons with accelerated maturation of glial lineages. These impairments were associated with deregulation in the expression profiles of pro-neural genes in dorsal and ventral forebrain-derived neural stem cell species. Taken together, these experimental observations suggest that H1 linker histone proteins are critical for the specification, maturation and fidelity of organ-specific cellular lineages derived from the three cardinal germ layers.

## Introduction

Eukaryotic DNA is packaged and maintained within highly regulated chromatin structures through association with various histone proteins. The fundamental unit of chromatin, the nucleosome, consists of ∼146 bp of DNA wrapped around an octamer of core histones, including H2A, H2B, H3 and H4 [1]. H1 linker histone proteins also represent an integral component of chromatin but share little conservation with other core histone members. H1 histones bind to linker DNA that exists between nucleosomes and contributes to the: 1) organization and stabilization of chromosomal DNA; 2) folding of nucleosome filaments into higher-order structures, and 3) modulation of gene transcription through limitation of nucleosome mobility and transcription factor access [2]–[6].

Mammals have at least eleven histone H1 variants, including somatic subtypes (H1a-H1e), testis-specific (H1t, H1T2 and HILS1) and oocyte-specific (H1oo) variants, and differentiation-associated (H1.0) subtypes, each exhibiting differential temporally and spatially defined expression profiles and locus-specific gene interactions [3]. Deletion of histone H1 in lower eukaryotes results in shortened life span and a variety of developmental defects in *A. Immersus* and *Arabidopsis*, respectively [7], [8]. In higher eukaryotes, deletion of histone H1.1, mouse testis-specific histone H1t, or individual deletions of murine somatic histone subtypes do not cause significant anomalies despite affecting specific gene expression in different cell types [9]–[11]. However, a 50% reduction of total H1 histone expression levels in mice as a result of concomitant deletion of three H1 histone subtypes (H1c, H1d and H1e) causes embryonic lethality as early as E8.5 with a wide range of developmental defects [12]. The precise developmental functions of these H1 linker histone subtypes, however, remains undefined.

Embryonic stem cells (ESCs) have the capability of giving rise to embryoid bodies comprised of cells representing the three cardinal germ layers and are useful tools to study developmental programs, which are otherwise relatively experimentally inaccessible in *in vivo* systems [13]. H1 linker histones have been found to be highly mobile in ESCs with subtypes H1c, H1d and H1e being most abundant [14], [15]. Depletion of H1c, H1d and H1e in ESCs (H1-KO ESCs) reduced chromatin compaction and caused alterations in specific gene expression profiles that are associated with modification of core histones and impairment in DNA methylation [14]. Recent studies by Fan et al. also demonstrated that H1-KO ESCs fail to properly form embryoid bodies (EBs), exhibit disruption of specific patterns of gene expression and fail to undergo neural differentiation [16]. However, the precise roles of H1 linker histone subtypes in these seminal developmental processes have not been fully examined.

In this study, we further examined the roles of H1 linker histone subtypes during early embryonic development. Utilizing established ESC culture paradigms, we analyzed the capacity of H1-KO ESCs to recapitulate early developmental events, including stem cell maintenance, the elaboration of the cardinal germ layers, and specification and maturation of organ-specific cellular lineages. Our studies demonstrate that H1 linker histones are essential for preserving the fidelity of mesoendodermal and neuroectodermal lineages, in part, by suppressing the generation of alternate cell fates.

## Results

### Absence of three H1 linker histone somatic variants in ESCs does not change the expression profiles of pluripotency markers but modulates cellular differentiation at the embryoid body stage

**Figure 1:**
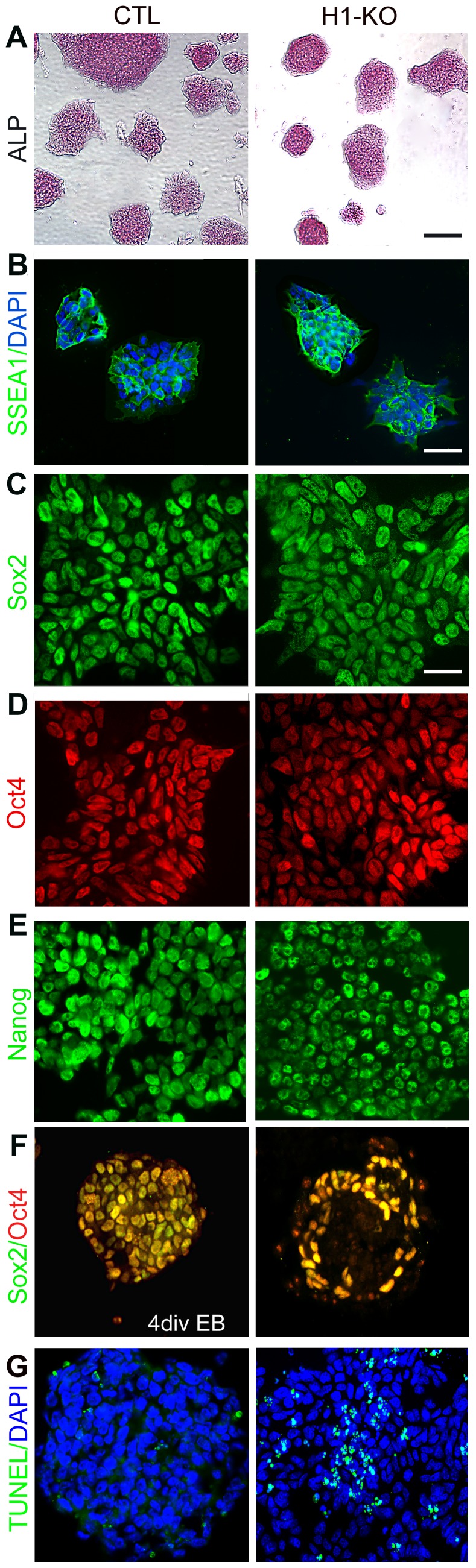
H1-KO ESCs maintain embryonic stem cell and pluripotency markers while exhibiting increased numbers of TUNEL+ cells during EB formation. (A–G) CTL and H1-KO ESC lines proliferating in the presence of LIF maintained expression of the embryonic stem cell markers, alkaline phosphatase (ALP) and SSEA1, as well as the pluripotency markers, Sox2, Oct4 and Nanog. (A–B, C–E). At 4 DIV H1-KO EBs exhibited statistically significant reductions in Sox2 and Oct4 (n = 2300, 2250 for CTL and H1-KO, respectively with a p<0.001) (F) and an increase in the number of TUNEL+ cells (n = 1308, 3127 for CTL and H1-KO, respectively with a p<0.0001) (G). Scale bar  =  (A) 500 µm; (B) 200 µm and (C–G) 20 µm.

Two H1-KO ESC lines (F6 and F1) and two control ESC lines (F18 and ATCC) were employed in our study. As the experimental observations are consistent, we will focus on data derived from the H1-KO (F6) and CTL (F18) cell lines, hereby referred to as H1-KO and CTL cells respectively. The H1-KO ESCs have been shown to exhibit normal growth rates and appear morphologically undifferentiated [14]. To more definitively characterize their cellular maintenance and lineage potential, H1-KO ESCs were initially examined for the expression of the ESC markers, alkaline phosphatase and SSEA1, and the core pluripotency markers, Sox2, Oct4, and Nanog. Immunofluorescence microscopy revealed that H1-KO ESC lines exhibited normal expression profiles for ESC and pluripotency markers as compared to CTL ESCs (Figure 1A–E). Experiments conducted utilizing ATCC and F1 lines yield comparable expression profiles of ESC and pluripotency markers (Figure S1A–E & Table S1). These results indicate that H1 linker histone subtypes, H1c, H1d and H1e are not essential for initial maintenance of ESC pluripotency and are consistent with previous studies examining the differentiation potential of these ESC lines [12] [15] [16].

**Figure 2:**
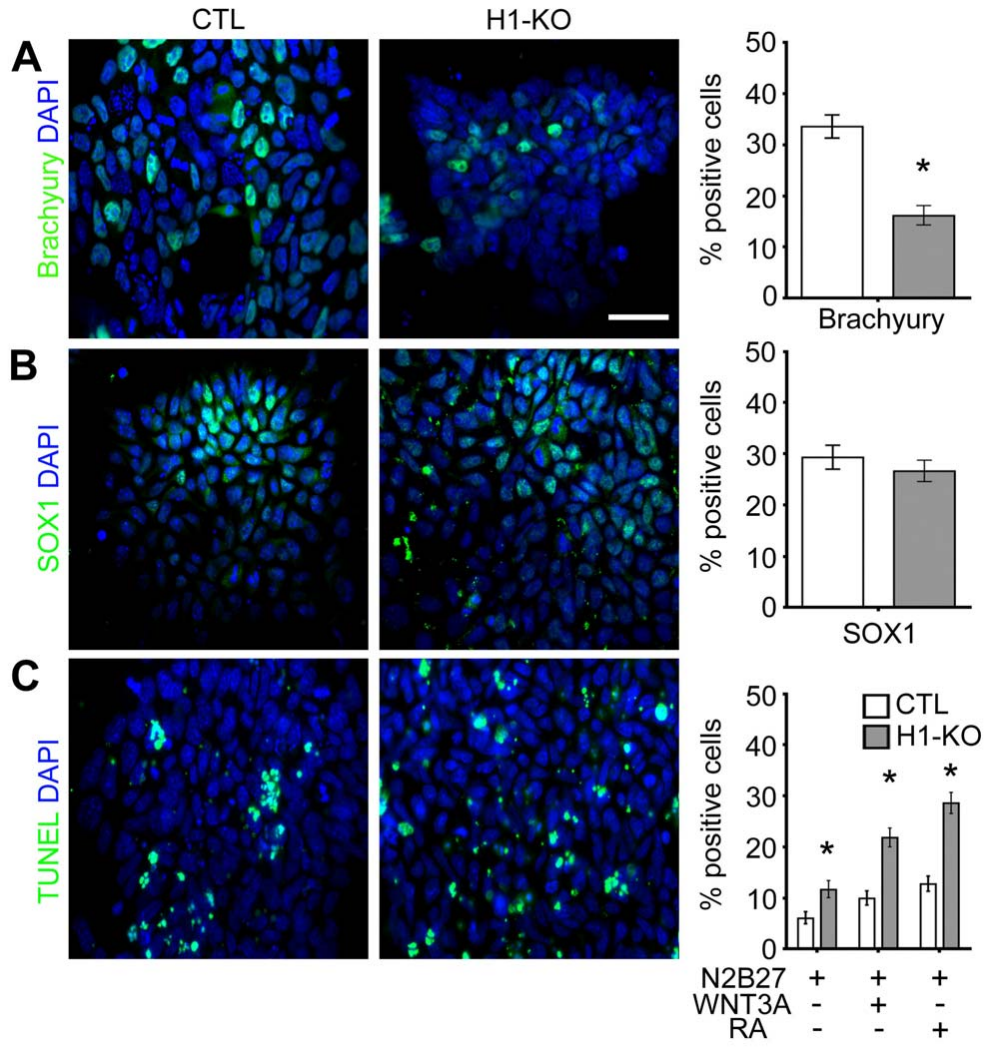
Deregulation of the induction and survival of mesendodermal and neuroectodermal cell types in H1-KO ESCs. (A) Representative images of immunofluorescence analysis of Brachyury+ mesendodermal progenitors after Wnt3A induction and the corresponding quantification of reduction in Brachyury+ cells in H1-KO cells (n = 1663, 1445 for CTL and H1-KO, respectively). (B) Representative images of immunofluorescence analysis of SOX1+ neuroectodermal progenitors after RA induction and the corresponding quantification of SOX1+ cells in H1-KO cells (n = 1462, 1722 for CTL and H1-KO respectively). (C) Representative images of immunofluorescence analysis of TUNEL+ cells and quantification of TUNEL+ in different conditions (Pre-induction: n = 1574, 1400; RA-induction: 1983, 1847; Wnt3A-induction: n = 1805, 1935; for CTL and H1-KO respectively). All error bars represent ±95% CI; * 0.0001 unless otherwise noted. Scale bar = 20 µm.

The findings that H1-KO ESCs retained normal profiles of markers of pluripotency and ESC identity suggested that the occurrence of developmental deficiencies in H1-KO mouse embryos as early as E8.5 may have arisen during germ layer specification and incipient organ-specific lineage elaboration. To begin to characterize these putative early developmental defects, H1-KO ESCs were first cultured in suspension to form EBs, which partially recapitulate the early embryonic developmental milestones associated with formation of the three cardinal germ layers (Flow Chart S1) [17], [18]. Immunofluorescence microscopy of 4 days *in-vitro* (DIV) EBs revealed that H1-KO EBs displayed statistically significant reductions in the expression patterns of Oct4 and Sox2 as compared to CTL EBs (Sox2: 96.43% vs. 74.57%, p-value<0.0001; Oct4: 94.70% vs. 76.93%, p-value<0.0001, CTL and H1-KO respectively; Figure 1F and Figure S1F & Table S1). This could be related to extensive cell death in H1-KO EBs as revealed by the presence of TUNEL+ cells, or to impaired differentiation into the three cardinal germ layers (TUNEL: 12.58% vs. 33.13%, p-value<0.0001; Figure 1G and Figure S1G & Table S1). As development ensues, mesendodermal progenitors give rise to definitive endoderm and mesoderm, whereas neuroectodermal progenitors give rise to the entire nervous system [19]. Thus, to investigate the role of H1 linker histone proteins in the elaboration of these cell types, we employed *in vitro* inductive paradigms using Wnt3A and retinoic acid (RA) to generate mesendodermal and neuroectodermal species, respectively [20]. H1-KO ESCs displayed a reduction in the percentage of Brachyury+ mesendodermal progenitors without a significant change in the percentage of SOX1+ neuroectodermal progenitors as compared to CTL ESCs (Brachyury: 16.12% vs. 33.55%, p-value<0.0001; SOX1: 26.60% vs. 29.27%, p-value = 0.0929; Figure 2A–B and Figure S2A–B). Furthermore, in concert with our observations during EB formation, H1-KO cells exhibited a significantly higher percentage of TUNEL+ dying cells prior to and following induction with Wnt3A and RA, as compared to CTL cells (Pre-Induction: 11.6% vs. 6.0%, p-value<0.0001; RA-induction: 21.8% vs. 9.9%, p-value<0.0001; Wnt3A-induction: 28.6% vs. 12.7%, p-value<0.0001; Figure 2C and Figure S2C). Consistent with previously published data, these observations indicate that H1 linker histone subtypes may be important for ESC-derived EB formation [16]. Moreover, they may have distinctive roles for the specification and survival of mesendodermal and neuroectodermal progenitors.

### Neural lineage differentiation is selectively deregulated in H1-KO ESCs

**Figure 3:**
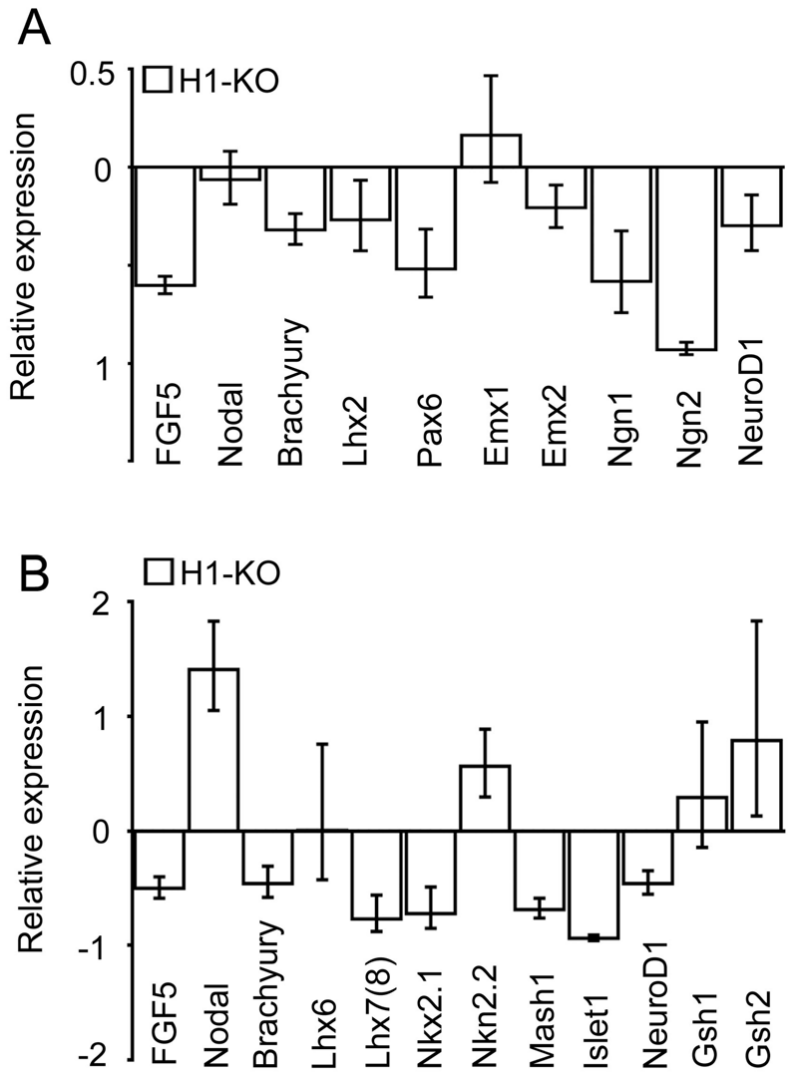
Comparative expression profiles of developmental genes in dorsal and ventral neural progenitor cell types derived from CTL and H1-KO ESCs using the ITFS/CM paradigm. (A) QPCR expression analysis of stage-specific developmental genes in dorsal neural progenitor cell types at 7 DIV. (B) QPCR expression analysis of stage-specific developmental genes in ventral neural progenitor cell types at 7 DIV. All error bars represent ±95% CI.

**Figure 4:**
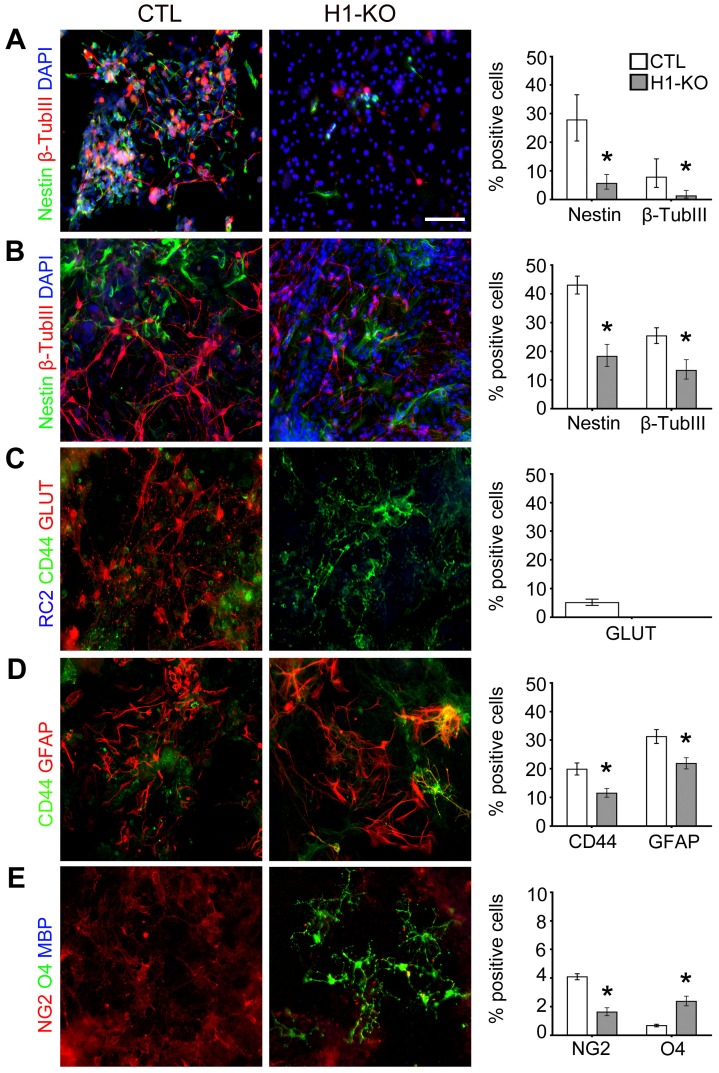
Selective developmental deregulation of H1-KO ESC-derived dorsal forebrain-specific neural lineage elaboration using the ITFS/CM paradigm. (A) At 7DIV, CTL cell lines displayed an increased number of nestin+ NSCs and β-tubulin III+ neuronal progenitor species compared to the H1-KO cell lines (n = 115, 319 for CTL and H1-KO respectively). (B) Following additional *in vitro* maturation, at 14 DIV CTL cell lines exhibited higher numbers of NSCs and neuronal progenitor species than H1-KO cell lines (n = 243, 389 for CTL and H1-KO respectively). (C) CTL cell lines showed a robust complement of glutamatergic neurons while H1-KO cell lines displayed an absence of normal neuronal differentiation at 21 DIV. (D) CTL and H1-KO cell lines showed similar profiles of GFAP+ astrocytes with a few CD44+ progenitors at 21 DIV (n = 1399, 1684 for CTL and H1-KO respectively). (E) The elaboration of O4+ oligodendrocyte progenitors in H1-KO cell lines was accelerated compared to those of CTL cell lines which predominantly has NG2+ oligodendrocyte precursors at 18 DIV (n = 3400, 800 for CTL and H1-KO respectively). All error bars represent ±95% CI, * p<0.05 unless otherwise noted. Scale bar = 100 µm.

Given the observations of atypical formation of EBs and regional cell death during germ layer elaboration of H1-KO ESCs, it is likely that additional specification and maturational processes associated with tissue-specific cellular lineages may also be adversely affected and may therefore contribute to the *in vivo* developmental defects and embryonic lethality previously observed. Ectoderm-derived neural differentiation was examined by utilizing an established ESC differentiation paradigm (EB/ITFS/CM), previously described by McKay et al. (1996) to generate neural stem cells (NSCs) and subsequently more differentiated neural lineage species [21]. As compared to CTL cells, expression analysis of H1-KO dorsally-derived NSCs revealed significant down regulation of a primitive ectodermal gene, *FGF5* (Fold change [Fc]: 0.398, p-value<0.001) a mesodermal gene, *Brachyury* (Fc: 0.681, p-value<0.001), dorsal patterning/arealization genes, *Lhx2* (Fc: 0.732, p-value = 0.001), *Pax6* (Fc: 0.418, p-value<0.001) and *Emx2* (Fc: 0.794, p-value = 0.01) as well as proneural/neuronal maturation genes *Ngn1* (Fc: 0.418, p-value<0.001), *Ngn2* (Fc: 0.069, p-value<0.001), and *NeuroD1* (Fc: 0.703, p-value<0.0001). There was no significant change in the expression of *Nodal* (Fc: 0.937, p-value = 0.067) that is present in endodermal lineages or of *Emx1*, (Fc: 1.164, p-value = 0.291) a homeodomain transcription factor involved in dorsal patterning (Figure 3A and Figure S3A & Table S1). Similarly, expression analysis of H1-KO ventrally-derived NSCs revealed down regulation of ectodermal, *FGF5* (Fc: 0.498, p-value<0.001) and mesodermal, *Brachyury* (Fc: 0.54, p-value<0.001) genes, ventral patterning/arealization genes, *Lhx7/8* (Fc: 0.231, p-value<0.001) and *Nkx2.1* (Fc: 0.277, p-value<0.001), and proneural/neuronal maturation genes, *Mash1* (Fc: 0.314, p-value<0.0001), *Islet1* (Fc: 0.062, p-value<0.0001) and *NeuroD1* (Fc: 0.54, p-value = 0.001). The expression of *Nodal* (Fc: 2.409, p-value<0.001) and *Nkx2.2* (Fc: 1.564, p-value<0.001), a homeodomain transcription factor involved in the elaboration of oligodendroglial cells, were upregulated whereas there was no significant change in the expression of other ventral patterning genes, including *Lhx6* (Fc: 1.003, p-value = 0.986), *Gsh1* (Fc: 1.292, p-value = 0.317) and *Gsh2* (Fc: 1.79, p-value = 0.063) (Figure 3B and Figure S3B & Table S1). Consistent with the significant impairment in the expression profiles of genes involved in patterning, specification, and maturation during early neural differentiation of H1-KO ESCs, immunofluorescence microscopy revealed significant reductions in the elaboration of nestin+ NSCs and early β-tubulin III+ neuronal lineage species from H1-KO ECSs as compared to CTL ESCs at both 7DIV and 14DIV(7DIV: Nestin: 5.6% vs. 27.8%, p-value<0.0001; β-tubulin III: 1.3% vs. 7.8%, p-value = 0.0013; Figure 4A and Figure S4A & Table S1; 14DIV: Nestin: 18.3% vs. 43.0%, p-value<0.0001; β-tubulin III: 13.4% vs. 25.4%, p-value<0.0001; Figure 4B and Figure S4B & Table S1). These findings indicate that H1c, H1d and H1e subtypes may play important roles in the temporal expression profiles of patterning, specification, and maturational genes resulting in selective alterations in the elaboration of NSCs and neural precursors.

**Figure 5:**
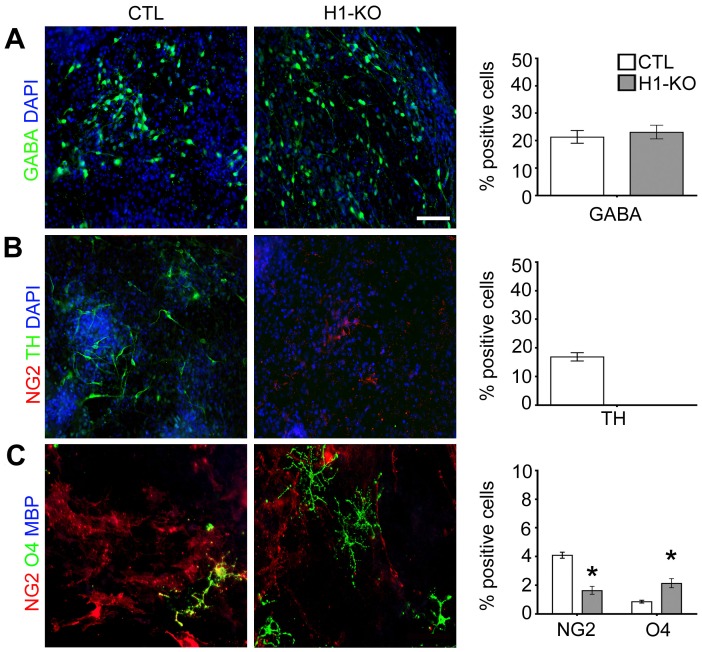
Selective developmental deregulation of H1-KO ESC-derived ventral brain-specific neural lineage elaboration using the ITFS/CM paradigm. (A) Both CTL and H1-KO cell lines exhibited comparable profiles of GABA+ GABAergic neurons (n = 959, 1102 for CTL and H1-KO respectively). (B) H1-KO cell lines do not give rise to TH+ dopaminergic neurons as compared to CTL cell lines. (C) The elaboration of O4+ oligodendrocyte progenitors in H1-KO cell lines was accelerated compared to those of CTL cell lines (n = 3400, 8000 for CTL and H1-KO respectively). All error bars represent ±95% CI, * p<0.05 unless otherwise noted. Scale bar = 100 µm.

Given our previous observations of impairments in the elaboration of H1-KO NSCs, we sought to further examine the fidelity of H1-KO ESC differentiation into the three principal neural cell types, neurons, astrocytes, and oligodendrocytes. Specific growth factor paradigms were utilized to partially recapitulate the *in vivo* spatial specification and maturation of neural lineage subtypes *in vitro*: dorsal telencephalic (glutamatergic neurons, astrocytes); ventral telencephalic (GABAergic neurons, oligodendrocytes); and ventral midbrain (dopaminergic neurons) (see *Materials and Methods* and Flow Charts S2–3). Using neuronal differentiation paradigms, we identified selective impairments in the specification and maturation of GLUT+ glutamatergic and TH+ dopaminergic neurons in H1-KO ESCs as compared to their corresponding CTL ESCs (Figure 4C and Figure 5B; Figure S4C and Figure S5B & Table S1), whereas the elaboration of GABAergic neurons was unaffected (GABA: 23.0% vs. 21.3%; p-value = 0.9098; Figure 5A). In addition, H1-KO ESCs gave rise to a reduced proportion of CD44+ astroglial precursors and GFAP+ astrocytes as compared to CTL ESCs (CD44: 11.5% vs. 19.9%, p-value<0.0001; GFAP: 21.9% vs. 31.2%, p-value = 0.0033; Figure 4D and Figure S4D & Table S1). By contrast, there was precocious elaboration of O4+ oligodendrocyte progenitors in H1-KO ESCs as compared to CTL ESCs, which contained predominantly NG2+ oligodendrocyte precursors (NG2: 1.6% vs. 4.1%, p-value<0.0001; O4: 2.4% vs. 0.7%, p-value<0.0001; Figure 4E and Figure S4E & Table S1). These observations suggest that H1c, H1d and H1e subtypes play important roles in the subsequent specification and maturation of selective regional neuronal and glial subtypes.

**Figure 6:**
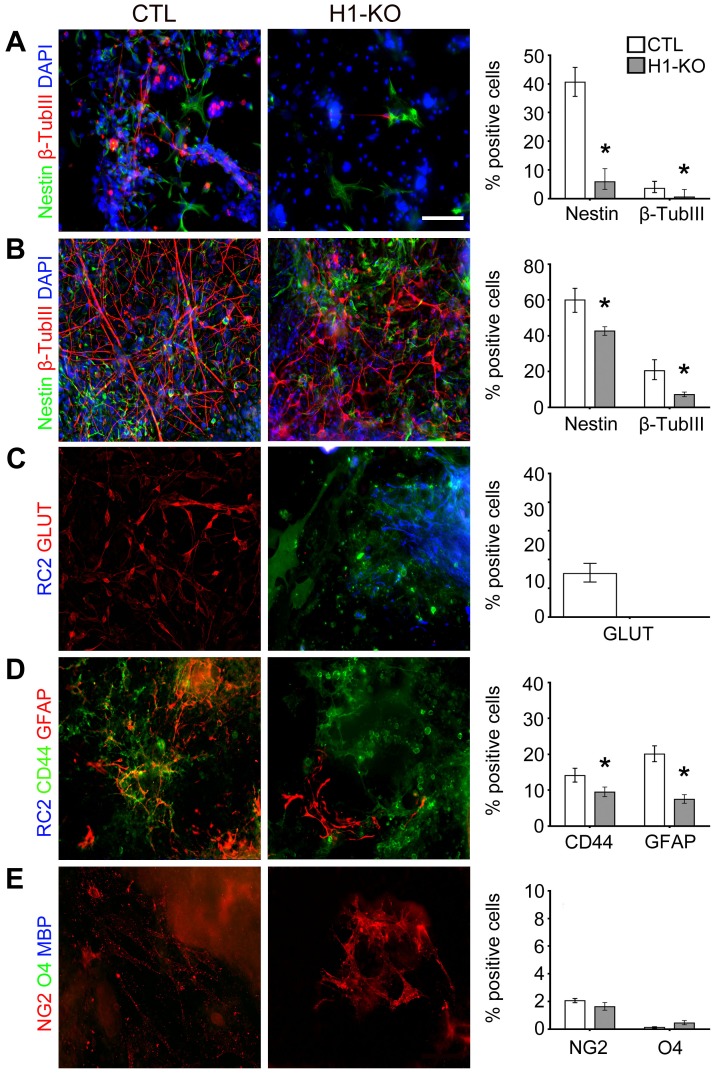
Selective developmental deregulation of H1-KO ESC-derived dorsal forebrain-specific neural lineage elaboration using the Neurobasal paradigm. (A) H1-KO cell lines generated significantly less nestin+ NSCs and β-tubulin III+ neuronal progenitor species as compared to CTL cell lines (n = 362, 170 for CTL and H1-KO respectively). (B) Following additional *in vitro* maturation, CTL cell lines generated higher numbers of nestin+ NSCs and β-tubulin III+ neuronal progenitor species than H1-KO cell lines (n = 200, 376 for CTL and H1-KO respectively). (C) H1-KO ESCs failed to give rise to glutamatergic neurons as compared to CTL ESCc. (D) H1-KO ESCs generated significantly lower number of CD44+ astrocytic precursors and GFAP+ astrocytes as compared to CTL ESCs (n = 1256, 1859 for CTL and H1-KO ESCs, respectively). (E) The elaboration of dorsally derived oligodendrocyte progenitors was low in both cell lines. All error bars represent ±95% CI, * p<0.05 unless otherwise noted. Scale bar = 100 µm.

**Figure 7:**
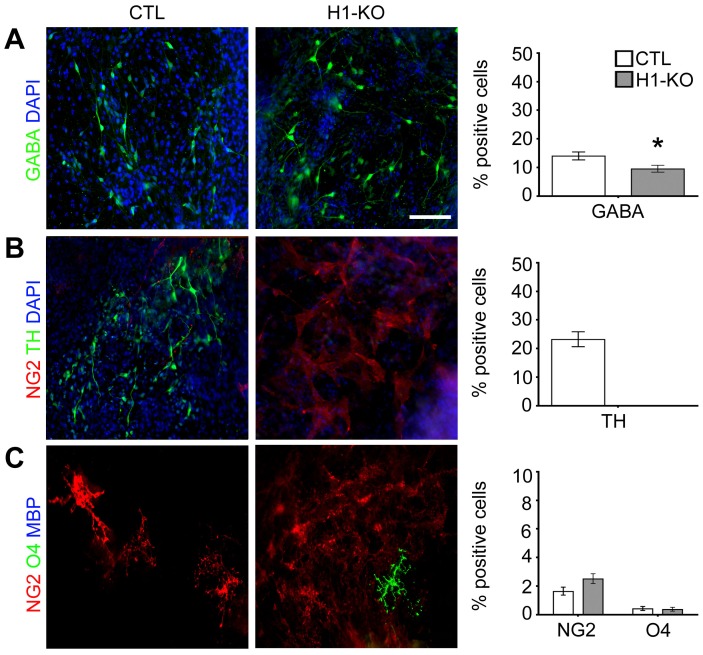
Selective developmental deregulation of H1-KO ESC-derived ventral brain-specific neural lineage elaboration using the Neurobasal paradigm. (A) There was a modest reduction in the percentage of GABAergic neurons derived from in H1-KO cell lines as compared to the CTL cell lines (n = 2393, 2265 for CTL and H1-KO respectively). (B) H1-KO cell lines did not give rise to TH+ dopaminergic neurons as compared to CTL cell lines. (C) The elaboration of ventrally-derived oligodendrocyte progenitors was low in both cell lines. All error bars represent ±95% CI, * p<0.05 unless otherwise noted. Scale bar = 100 µm.

To further validate and define the significance and specificity of the previous findings, a selective neuronal differentiation paradigm was employed [22], [23]. This complementary clonal differentiation paradigm favors neuronal over glial subtype-specific lineage elaboration through the use of Neurobasal media supplemented with B27 that has been shown to preferentially support neuronal growth and maturation [24]. In concert with findings obtained from the previous EB/ITFS/CM paradigm, the percentage of nestin+ NSCs and β-tubulin III neuronal species was significantly reduced in H1-KO cells when compared to CTL cells at 7 DIV (Nestin: 5.9% vs. 40.6%, p-value<0.0001; β-tubulin III: 0.6% vs. 3.4%, p-value = 0.045; Figure 6A and Figure S6A & Table S1) and remained significantly lower at 14DIV (Nestin: 42.6% vs. 60.0%, p-value<0.0001; β-tubulin III: 7.2% vs. 20.5%, p-value<0.0001; Figure 6B and Figure S6B & Table S1). Moreover, the generation of dorsal forebrain-derived GLUT+ glutamatergic neurons and ventral midbrain-derived TH+ dopaminergic neurons was completely absent in the H1-KO cells, despite being subjected to a more favorable culture condition for induction of neurogenesis (Figure 6C and Figure 7B; Figure S6C and Figure S7B & Table S1). Ventral forebrain-derived GABAergic neuron elaboration was significantly impaired in H1-KO cells as compared to CTL cells (GABA: 9.5% vs. 14.0%, p-value<0.0001; Figure 7A and Figure S7A & Table S1). The percentage of CD44+ astrocyte precursors and GFAP+ astrocytes was significantly reduced in H1-KO cells as compared to CTL cells (CD44: 9.5% vs. 14.1%, p-value<0.0001; GFAP: 7.5% vs. 20.1%, p-value<0.0001; Figure 6D and Figure S6D & Table S1). Although very few NG2+ oligodendrocyte precursors were specified in both CTL and H1-KO cell lines, the maturation of O4+ oligodendrocyte progenitors was not sustainable under these media conditions (Figure 6E and Figure 7C; Figure S6E and Figure S7C & Table S1). These cumulative findings from the second distinct neural differentiation paradigm are consistent with our previous findings of selective impairments in the specification and maturation of glutamatergic and dopaminergic neurons as well as astroglial species.

### Aberrant cardiomyocyte and hepatic differentiation of H1-KO ESCs

**Figure 8:**
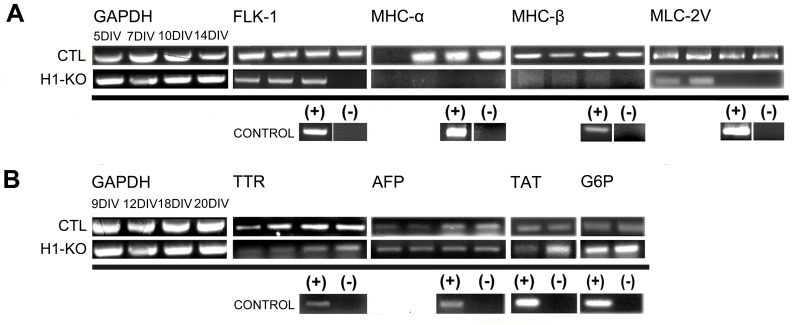
Comparative developmental gene expression profiles associated with cardiomyocyte and hepatic differentiation of CTL and H1-KO ESCs. (A) *Cardiomyocyte differentiation*. CTL cell lines displayed normal acquisition of cardiomyocyte marker genes FLK-1, MHC-α, MHC-β, and MLC-2V, whereas the expression of these genes was significantly developmentally deregulated in the H1-KO cell lines. (B) *Hepatic differentiation*. The developmental profiles of gene expression of TTR, AFP, TAT and G6P were similar between CTL and H1-KO cell lines.

The various embryonic developmental abnormalities observed in the homozygous H1-KO mice occurring as early as E8.5 may be due to the aberrant specification and maturation of organ-specific cellular lineages derived from the three cardinal germ layers. Given that the integrity of mesendodermal progenitor specification has been shown to be impaired, the fidelity of mesodermal induction and organ-specific lineage potential was next examined by monitoring the differentiation of the H1-KO ESCs to cardiomyocytes using a previously established paradigm [25]. We performed our developmental characterization using markers of vascular endothelial growth factor receptor-2, FLK-1 [26], and the expression of the sarcomeric proteins, MHC-α, MHC-β and MLC-2V, which define progressive stages of mesodermal induction and cardiomyocyte specification and maturation *in vitro* [27]. The expression profiles of FLK-1, MHC-α, MHC-β and MLC-2V have been documented starting at 5 DIV for wild-type cell lines [30]. It is noteworthy that gene expression analysis using RT-PCR revealed that CTL cells followed the normal program of progressive cardiomyocyte developmental gene expression as previously described [25], [27] (Figure 8A and Figure S8A). However, the induction of MHC-α and MHC-β transcripts was impaired in H1-KO ESCs (Figure 8A and Figure S8A). Moreover, H1-KO ESCs exhibited reduced expression of FLK-1 and of MLC-2V during later stages of differentiation (Figure 8A and Figure S8A). These observations indicate that H1c, H1d and H1e subtypes may play important developmental roles in the specification and maturation of mesoderm-derived cardiomyocytes.

We next investigated the integrity of endodermal induction and endoderm-derived tissue-specific differentiation utilizing specific paradigms to generate hepatic [28] and pancreatic [29] cellular lineages. The progressive developmental gene expression profiles for hepatic differentiation *in vitro* encompass the expression of transthyretin (TTR) and alpha-fetoprotein (AFP), which begins as early as 9 DIV and persists until at least 20 DIV, followed by the expression of tyrosine aminotransferase (TAT) and glucose-6-phosphate (G6P) that are restricted to temporal intervals around 18 DIV [28]. Gene expression analysis using RT-PCR revealed that hepatic differentiation in CTL cells followed the appropriate gene expression profiles, and the differentiation of H1-KO ESCs also exhibited relatively normal patterns of hepatic gene expression (Figure 8B and Figure S8B). These observations indicate that H1c, H1d and H1e subtypes may not be required for orchestrating proper gene expression associated with hepatic differentiation from ESCs.

### Ectopic neurogenesis and cardiomyogenesis during pancreatic differentiation of H1-KO ESCs

**Figure 9:**
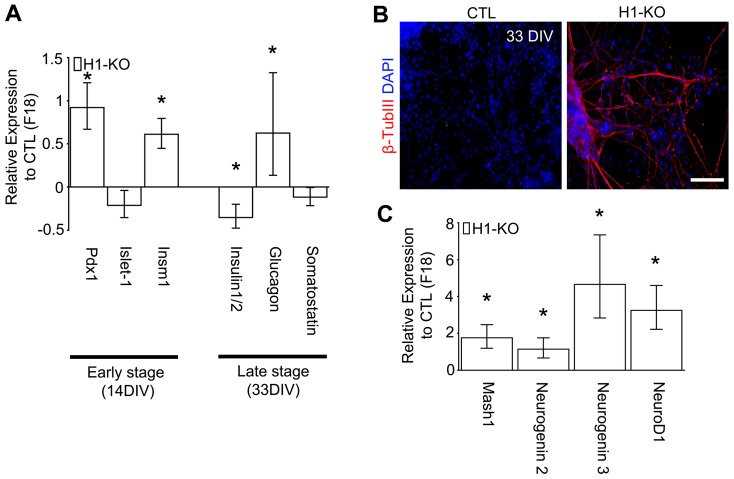
Ectopic neurogenesis associated with pancreatic differentiation paradigms in H1-KO ESCs. (A) Gene expression analysis of pancreatic genes in early pancreatic progenitors and late mature pancreatic cells in H1-KO cell lines. (B) At 33 DIV, H1-KO cell lines ectopically generated β-TubIII+ neurons. (C) Gene expression analysis of proneural genes in early pancreatic progenitors in H1-KO cell lines as compared to CTL cell lines. All error bars represent ±95% CI; *p<0.0001 unless otherwise noted. Scale bar = 20 µm.

In addition to hepatic cells, pancreatic endocrine cells are also derived from the endoderm [30]. A previous report defined the developmental gene expression program during pancreatic differentiation *in vitro* that includes sequential expression of early pancreatic progenitor genes, Pdx1, Islet-1, Insm1, followed by mature pancreatic genes, Insulin1/2, Glucagon and Somatostatin [29]. Herein, we found increases in the expression of Pdx1 (Fc: 1.921, p-value<0.0001) and Insm1 (Fc: 1.612, p-value<0.0001) but not Islet-1 (Fc: 0.788, p-value = 0.995) during early pancreatic differentiation (Figure 9A and Figure S9A & Table S1). By contrast, during later stages of pancreatic cellular maturation, we found selective reduction in the expression of Insulin1/2 (Fc: 0.647, p-value<0.0001) as compared to CTL ESCs (Figure 9A and Figure S9A & Table S1). Additionally, robust profiles of β-tubulin III+ neurons were evident in late-stage pancreatic differentiation cultures of H1-KO cells (Figure 9B and Figure S9B & Table S1). This phenotype was corroborated by an increase in the expression of the proneural genes, *Mash1* (Fc: 2.762, p-value<0.0001), *Neurogenin 2* (Fc: 2.143, p-value = 0.002), *Neurogenin 3* (Fc: 5.658, p-value<0.0001), as well as *NeuroD1* (Fc: 4.249, p-value<0.0001) at early stages of pancreatic differentiation (Figure 9C), and of the *Neurofilament protein heavy-chain subunit* (Fc: 1.35, p-value = 0.009) at later differentiation stages (data not shown). In addition, we also observed ectopic cardiomyogenesis in one of the H1-KO ES cell lines (Figure S10). These cumulative findings further suggest that the presence of H1 linker histone subtypes H1c, H1d and H1e, in ESCs is essential for maintaining the integrity of endodermal germ layer induction and associated lineage differentiation, in part, by active suppression of alternative germ layers, organ-specific lineage specification and maturation programs.

## Discussion

In this study, we have shown that the three H1 linker histone subtypes, H1c, H1d and H1e are important for the differentiation and survival of mesendodermal and neuroectodermal lineages. In this regard, we have demonstrated that these H1 linker histone subtypes are involved in the elaboration of cardiomyocyte and pancreatic cell types, and in the suppression of alternate germ layer-derived organ-specific cell types during the process of pancreatic differentiation. In addition, our studies reveal that H1 linker histones subtypes play important roles in the specification and maturation of neural stem and progenitor cells and specific neuronal and glial cell types.

The initial observation that the H1-KO ESCs appeared morphologically normal and maintained expression of key pluripotency markers is not surprising given the fact that chromatin binding of H1 linker histone proteins is highly dynamic in ESCs compared to lineage-specific differentiated progeny [15], [31], [32]. The ‘hyperdynamic’ nature of histones in ESCs allows for an “open” chromatin structure that is permissive for transcriptional activation [33], [34]. This suggests that the full functional complement of H1 linker histone subtypes, commonly known to bind and stabilize higher-order chromatin structure, may not be as essential in ESCs as in other cell types [35], [36]. In addition, previous studies performed with these cell lines suggest that cellular homeostasis of H1-KO ESCs may be maintained by the compensatory effects of the remaining H1 linker histone subtypes, namely H1a, H1b and H1.0, whose expression has been shown to be significantly up regulated in ESCs [12] [16]. Moreover, non-histone mechanisms in ESCs may play additional roles in chromatin structural maintenance, thereby allowing the redeployment of H1 linker histone proteins to other later developmental functions. One mechanism, in particular, encompasses the binding of Oct4, Nanog and other pluripotency factors to DNase I hypersensitivity sites (HS sites) within the Nanog locus, resulting in chromatin restructuring and modulation of Nanog-associated genes linked to pluripotency and early development, including Dpp3 and GDF3 [35], [37].

Significant developmental abnormalities are uncovered when H1-KO ESCs were induced to differentiate into the three cardinal germ layers [16]. The dependence of ESC differentiation on the basal state of H1 linker histone proteins has also been demonstrated in a previous study in which constitutive binding of a mutant form of H1 abolished ESC-mediated neural differentiation [15]. Consistent with this observation, our study revealed deregulated profiles of neuroectodermal differentiation in H1-KO ESCs, including aberrant specification of NSCs and early neuronal precursors. Interestingly, utilization of specific *in vitro* ESC culture paradigms developed in our laboratory [38], [39] revealed that with proper growth factors supplementation H1-KO cell lines can generate forebrain-specific neuronal and glial species. These alterations in the profiles of neural differentiation may also be indicative of impaired mesodermal induction, because mesoderm has important instructive roles in the development and patterning of the nervous system [40]. Moreover, deregulated expression profiles for genes involved in endoderm-derived pancreatic but not hepatic lineage elaboration suggested that H1 histones are also required to ensure the presence of stable transcriptional programs during specification and maturation of this tissue-specific cell type. During embryogenesis, pancreatic lineages are formed from mesendoderm-derived definitive endoderm through a default pathway, whereas hepatic lineages require fibroblast growth factor and bone morphogenetic protein inductive signals emanating from mesoderm with concurrent repression of pancreatic lineages [41]. In addition, previous studies performed with these cell lines suggest that cellular homeostasis of H1-KO ESCs may be maintained by the compensatory effects of the remaining H1 linker histone subtypes, namely H1a, H1b and H1.0, whose expression has been shown to be significantly up regulated in ESCs [12] [16]. However, our results suggest that beyond the EB differentiation stage, selective upregulation of other somatic H1 linker histone subtypes may not be necessary to support the elaboration of tissue specific lineages. Interestingly, we observed ectopic neurogenesis and cardiomyogenesis in conditions favoring pancreatic differentiation from H1-KO ESCs. These observations suggest that H1 linker proteins may modulate the context-specific effects of mesoderm on the induction of cardiomyocytes and neural lineage cell types as well as the repression of pancreatic lineage elaboration.

Alternatively, the observed impairment in cardiomyocyte differentiation in H1-KO ESCs may be accounted for by compensatory up regulation of H1b, which results in ectopic binding to Msx1 and aberrant repression of MyoD expression associated with impairments in myogenic differentiation programs [42]. Additionally, these developmental impairments may also be reflective of alterations in the expression of Nedd9, previously shown to be selectively reduced in H1 KO ESCs. Nedd9 is an adhesion and adaptor molecule involved in modulating the elaboration of committed neuronal and glial subtypes, as well as progenitors of other tissues including gut, respiratory epithelium, kidney and cardiac muscle [14], [43].

Stoichiometric relationships are also known to play an important role in H1 histone linker functions. In this regard, non-physiological increases in expression levels of H1 linker histone subtypes H1a and H1b in H1-KO ESCs may, in part, explain impairments in the elaboration of tissue-specific lineages [12], [14]. Indeed, it has been shown that in kidney, liver, lung and brain development H1a and H1b subtypes are progressively lost, whereas H1c and H1e represent the most prominent H1 linker subtypes [44]–[46]. This possibility is supported by the fact that different H1 linker histone subtypes are involved in distinct biological functions, differential chromatin binding affinities and in interaction with alternate networks of binding proteins in individual cell types [47]–[50].

Overall, our findings suggest that H1 linker histone proteins play important as well as previously unanticipated roles during the ESC maintenance and tissue-specific lineage elaboration. It is therefore necessary to further clarify the implications of our experimental observations with additional *in vivo* studies to more fully elucidate the mechanisms of action of individual H1 histone subtypes. These new initiatives will provide a more refined understanding of the precise roles of H1 linker histones during these seminal developmental processes.

## Materials and Methods

### Embryonic Stem Cell Models and Culture Paradigms

CTL (F18) and H1-KO (F6) ESC lines were previously generated by Fan et. al., [12], [14]. The number of ESC passages was kept at 8 passages or less and karyotypic profiles were analyzed prior to experimental manipulations. ESCs were maintained in an undifferentiated state on 0.1% gelatin-coated tissue culture plates in ES cell media consisting of knockout Dulbecco's minimal essential medium (Invitrogen, DMEM, 10313), 10% ES-qualified FBS (ATCC, SCRR-30-2020), 1X MEM nonessential amino acids (from 100× stock, Invitrogen 11140), 1X L-glutamine and antibiotics (from 100× stock, Invitrogen 10378-016), 0.1 mM 2-mercaptoethanol (Sigma, M7522), supplemented with 1000 U/ml of leukemia inhibitory factor (LIF/ESGRO; Chemicon, ESG1106). Media was changed daily and cell density was kept below 70% confluency.

### Germ Layer Progenitor Cell Differentiation and Embryoid Body Formation

Embryoid body differentiation was carried out by 1) plating ESCs onto non-adherent bacterial culture dishes at a density of 2–2.5×10^4^ cells/cm^2^, or 2) ESCs cultured as hanging drops [51] at a density of ∼400 cells per drop of 20 µl–30 µl ESC media, for 4 DIV. Differentiation of mesendodermal and neuroectodermal progenitors from ESCs were carried out as previously described [20]. Briefly, ESCs were plated at 1.5×10^4^ cells/cm^2^ on gelatin-coated tissue culture plates or poly-L-ornithine coated glass coverslips in N2B27 media for 48 hr (Pre-induction stage). After 48 hr, media is replaced with fresh N2B27 supplemented with either 500 nM retinoic acid (Sigma) or 200 ng/ml Wnt3A (R&D), and cells were cultured for a further 24–36 hr (Post-induction stage).

### Neuronal and Glial Differentiation – ITFS/CM Paradigm

The ITFS/CM neural differentiation includes selection and expansion of nestin-positive cells as previously described [21], followed by differentiation of specific neural lineages for glutamatergic neurons [52], GABAergic neurons [52], [53], astrocytes [52], and oligodendrocytes [52]. Briefly, ESCs were cultured in suspension cultures to form EB for 4 DIV. The EBs were then plated onto adhesive tissue culture surfaces and cultured for 6–8 DIV in ITFS medium consisting of DMEM/F12 (Invitrogen, 11330) supplemented with 5 µg/ml insulin (Sigma, I6634), 50 µg/ml transferrin (Sigma, T1147), 30 nM sodium selenium (Sigma, S5261), 5 µg/ml fibronectin (Sigma, F1141), and 1× L-glutamine and antibiotics (100× stock, Invitrogen 10378-016) to select for nestin-positive cells. After selection, cells were dissociated with 0.05% trypsin/0.04% EDTA and re-plated onto either tissue culture plates or glass coverslips pre-coated with poly-DL-ornithine/laminin (Sigma P8638; BD 354232) at a concentration of 1.5–2×10^5^ cells/cm^2^ in Complete Media (CM) (consisting of 500 ml DMEM/F12 supplemented with 1× N-2 (100× stock, Gibco, 17502-048) and 10 mg Insulin), supplemented with 1 mg/ml laminin (BD, 354232) and 10 ng/ml FG2 (BD, 354060) to expand cells for 4–6 DIV. After expansion, specific neural differentiation was induced by removal of FGF2 and supplemented with the following growth factors for a further 7 DIV: 1) glutamatergic neurons; 1000 U/ml LIF, 2) astrocytes; 20 ng/ml BMP2+1000 U/ml LIF, 3) GABAergic neurons; 100 ng/ml SHH + 10 µg/ml BMP2, 4) dopaminergic neurons; 100 ng/ml+100 ng/ml FGF8, 5) oligodendrocytes; 100 ng/ml SHH+10 ng/ml PDGFα.

### Neuronal and Glial Differentiation – NeuroBasal Paradigm

The differentiation protocol was carried out as previously described with minor modifications [22]. Briefly, ESCs were plated and grown on tissue culture plates coated with gelatin at a density of 1–1.5×10^4^ cells/cm^2^ in DMEM/F12 supplemented with N2 and Neurobasal supplemented with B27 in a (1∶1) ratio (all reagents are from Gibco). After 5–7 DIV, cells were dissociated with trypsin and re-plated onto glass cover slips coated with polyornithine/laminin and expansion of nestin-positive cells was performed using 10 ng/ml bFGF+1 µg/ml laminin. Induction of specific neural differentiation was performed as described in previously.

### Hepatic Differentiation

The hepatic differentiation paradigm was carried as previously described [28]. Briefly, ESCs (day 0) were maintained in suspension cultures to form EBs for 5 days in Iscove's modified DMEM (Invitrogen, 21056-023) supplemented with 20% NCS, 1X MEM nonessential amino acids, 1X L-glutamine and antibiotics and 0.1 mM 2-mercaptoethanol. On day 5, the EBs were plated onto collagen-I (BD, 354236)-coated tissue culture plates to allow for cell growth. On day 9, the media was supplemented with 100 ng/ml acidic fibroblast growth factor (aFGF, R&D, 232-FA) for another 3 days. On days 12–20, the media was supplemented with 20 ng/ml hepatocyte growth factor (HGF, R&D, 2207-HG). From days 15–20, the media was also supplemented with 10 ng/ml oncostatin-M (OSM, R&D, 495-MO), 10^−7^ M Dexamethasone (Sigma, D4902), 5 mg/ml insulin (Sigma, I6634), 5 mg/ml transferrin (Sigma, T1147), and 5 µg/ml sodium selenium (Sigma, S5261). The media was changed daily. Cells were analyzed at 12 days and 20 days for early hepatic progenitors and mature hepatocytes, respectively.

### Pancreatic Differentiation

The pancreatic differentiation paradigm was carried out as previously described [29]. Briefly, ESCs were grown to form EBs for 5 days in Iscove's modified DMEM supplemented with 20% NCS, 1X MEM nonessential amino acids, 1X L-glutamine and antibiotics and 0.1 mM 2-mercaptoethanol. After 5 DIV, EBs were plated onto gelatin-coated plates and allowed to expand for an additional 9 DIV. Thereafter, cells were dissociated using 0.05% trypsin/0.04% EDTA into single cells/small clusters and re-plated onto tissue culture plates pre-coated with Collagen I (Becton Dickinson, 4236) for an additional 19 DIV in defined medium consisting of DMEM/F12 (Invitrogen, 11330) supplemented with 20 nM progesterone (Sigma, P7556), 100 µM putrescine (Sigma, P5780), 1 µl/ml laminin (BD, 354232), 10 mM nicotinamide (Sigma, N3376), 25 µg/ml insulin (Sigma, I6634), 30 nM sodium selenium (Sigma, S5261), 50 µg/ml transferrin (Sigma, T1147), B-27 media supplement (Invitrogen, 17504-044) and 1X L-glutamine and antibiotics (from 100× stock, Invitrogen 10378-016). The media was changed every two days. Cells were collected at 21 and 33 DIV for committed pancreatic progenitors and mature pancreatic species, respectively.

### Cardiomyocyte Differentiation

Cardiomyocyte differentiation was carried out as previously described [25]. Briefly, ESCs were maintained in suspension cultures to form EBs for 4 DIV in knockout Dulbecco's minimal essential medium supplemented 10% NCS, 1X MEM nonessential amino acids, 1X L-glutamine and antibiotics and 0.1 mM 2-mercaptoethanol. Thereafter, EBs were plated onto gelatin-coated tissue culture plates and propagated for an additional 20 DIV until contractile cardiomyocytes were observed. Cells were harvested for mRNA and QPCR gene expression analysis at 4 DIV and 8 DIV, and 12 DIV for early and late cardiomyocyte progenitors, and for mature contractile cardiomyocytes, respectively.

### Immunohistochemistry Analysis and TUNEL Assay

Samples were fixed with 4% PFA and immunofluorescence analysis was carried out as previously described [38], [39]. Refer to Table S1 for the antibodies employed. TUNEL analysis was performed according to the manufacturer's protocols (Roche, 1168479591) and as previously described [39].[39].

### Quantitative Real-Time PCR (QPCR) Analysis

Harvesting of RNA from samples was carried out using TRI reagent (Molecular Research Center Inc, Cincinnati, OH, USA) according to manufacturer's protocol. Quantification of total RNA concentration was determined using the Qubit RNA assay kit and Qubit 2.0 Fluorometer (Invitrogen). Single strand cDNA synthesis was performed using the High Capacity cDNA Reverse Transcription Kit (Applied Biosystem, 4368814) following the manufacturer's recommendations. TaqMan probes and SYBR Green primers were purchased from PE Applied Biosystems and Invitrogen service respectively (See Table S2). We utilized either TaqMan Universal PCR Master Mix or SYBR Green Master Mix and ran samples in triplicate in the Model 7000 Real Time PCR system (Applied Biosystems, CA, USA). We chose the housekeeping gene hypoxanthine guanine phosphoribosyl transferase 1 (*HPRT1*). All RNA samples were normalized using fluorometric procedures and levels of cDNA input were quantified and normalized using the Qubit-Invitrogen fluorometric kit for single strand DNA (previous to this quantification, heteroduplex cDNA-mRNA were treated with RNAse H). Finally, an additional normalization step was employed through quantifying the ROX fluorescence emission in each well by multiplex scanning after each PCR cycle. These additional normalization procedures significantly contributed to the achievement of a uniform, and highly replicable data results across the technical and biological replicates. Data collection and quality assessment were performed utilizing 7000 SDS 1.1 RQ Software (Applied Biosystems, CA, USA). The statistical analysis was performed using the *Pair Wise Fixed Reallocation Randomization* test provided by the Relative Expression Software Tool (REST) developed by Corbett Research [54], [55]. Gene expression levels were reported using the relative RQ values ± 95% Confidence Interval (CI).

### Statistical Analysis

All quantifications of different culture models reflect the analysis of a minimum of 10 randomly selected fields from three distinctive biological replicates. Cell counts were performed using a 40× objective. Statistical comparisons were evaluated based on the type of data studied: proportions were compared with Chi-square test and the means of samples with either Mann-Whitney U test or *t*-test. Unless otherwise indicated, statistically significant differences between samples were considered using a probability of at least <0.0001 and/or <0.001.

## Supporting Information

Figure S1 **H1-KO (F1) ESCs maintain embryonic stem cell and pluripotency markers while exhibiting increased numbers of TUNEL+ cells during EB formation.** (A–G) CTL (ATCC) and H1-KO (F1) ESC lines maintained expression of the embryonic stem cell markers, ALP and SSEA1, as well as the pluripotency markers, Sox2, Oct4 and Nanog (A–B, C–E). At 4 DIV, H1-KO EBs exhibited statistically significant reductions in Sox2 and Oct4 (n = 2000, 2030 for CTL (ATCC) and H1KO(F1), respectively with a p<0.001) (F) and an increase in the number of TUNEL+ cells (n = 4140, 4730 for CTL (ATCC) and H1-KO (F1), respectively, p<0.0001) (G). Scale bar = (A) 500 µm; (B) 200 µm and (C–G) 20 µm.
(TIF)

Figure S2 **Deregulation of the induction and survival of mesendodermal and neuroectodermal cell types in H1-KO (F1) ESCs.** (A) Representative images of immunofluorescence analysis of Brachyury+ mesendodermal progenitors after Wnt3A induction and the corresponding quantification of reduction in Brachyury+ cells in H1-KO (F1) cells (n = 2065, 1359 for CTL (ATCC) and H1-KO (F1), respectively). (B) Representative images of immunofluorescence analysis of SOX1+ neuroectodermal progenitors after RA induction and the corresponding quantification of SOX1+ cells in H1-KO (F1) cells (n = 2149, 1713 for CTL (ATCC) and H1-KO (F1) respectively). (C) Representative images of immunofluorescence analysis of TUNEL+ cells and quantification of TUNEL+ in different conditions (Pre-induction: n = 3000, 1315; RA-induction: 2149, 1796; Wnt3A-induction: n = 2065, 1740; for CTL (ATCC) and H1-KO (F1) respectively). All error bars represent ±95% CI; *p<0.0001 unless otherwise noted. Scale bar = 20 µm.
(TIF)

Figure S3 **Comparative expression profiles of developmental genes in dorsal and ventral neural progenitor cell types derived from CTL (ATCC) and H1-KO (F1) ESCs using the ITFS/CM paradigm.** (A) QPCR expression analysis of stage-specific developmental genes in dorsal neural progenitor cell types at 7 DIV. (B) QPCR expression analysis of stage-specific developmental genes in ventral neural progenitor cell types at 7 DIV. All error bars represent ±95% CI.
(TIF)

Figure S4 **Selective developmental deregulation of H1-KO (F1) ESC-derived dorsal forebrain-specific neural lineage elaboration using the ITFS/CM paradigm.** (A) CTL cell lines displayed an increased number of nestin+ NSCs and β-tubulin III+ neuronal progenitor species compared to the H1-KO cell lines (n = 64, 300 for CTL (ATCC) and H1-KO (F1) respectively). (B) Following additional *in vitro* maturation, CTL (ATCC) cell lines exhibited higher numbers of NSCs and neuronal progenitor species than H1-KO (F1) cell lines (n = 962, 813 for CTL (ATCC) and H1-KO (F1) respectively). (C) CTL (ATCC) cell lines showed a robust complement of glutamatergic neurons while H1-KO (F1) cell lines displayed an absence of normal neuronal differentiation. (D) CTL (ATCC) and H1-KO (F1) cell lines showed similar profiles of GFAP+ astrocytes (n = 703, 611 for CTL (ATCC) and H1-KO (F1) respectively), but H1-KO (F1) gave rise to significantly decreased number of CD44+ progenitors (n = 673, 58 for CTL (ATCC) and H1-KO. (E) The elaboration of O4+ oligodendrocyte progenitors in H1-KO (F1) cell lines was accelerated compared to those of CTL (ATCC) cell lines which predominantly has NG2+ oligodendrocyte precursors (n = 400, 1390 for CTL (ATCC) and H1-KO F1) respectively). All error bars represent ±95% CI, * p<0.05 unless otherwise noted. Scale bar = 100 µm.
(TIF)

Figure S5 **Selective developmental deregulation of H1-KO (F1) ESC-derived ventral brain-specific neural lineage elaboration using the ITFS/CM paradigm.** (A) Both CTL (ATCC) and H1-KO (F1) cell lines exhibited comparable profiles of GABA+ GABAergic neurons (n = 1184 1257 for CTL (ATCC) and H1-KO (F1) respectively). (B) H1-KO (F1) cell lines do not give rise to TH+ dopaminergic neurons as compared to CTL cell lines. (C) The elaboration of O4+ oligodendrocyte progenitors in H1-KO (F1) cell lines was accelerated compared to those of CTL cell lines (n = 800, 3400 for CTL (ATCC) and H1-KO (F1) respectively). All error bars represent ±95% CI, * p<0.05 unless otherwise noted. Scale bar = 100 µm.
(TIF)

Figure S6 **Selective developmental deregulation of H1-KO (F1) ESC-derived dorsal forebrain-specific neural lineage elaboration using the Neurobasal paradigm.** (A) H1-KO (F1) cell lines generated significantly less nestin+ NSCs and β-tubulin III+ neuronal progenitor species as compared to CTL cell lines (n = 239, 238 for CTL (ATCC) and H1-KO (F1) respectively). (B) Following additional *in vitro* maturation, CTL cell lines generated higher numbers of nestin+ NSCs and β-tubulin III+ neuronal progenitor species than H1-KO cell lines (n = 357, 910 for CTL (ATCC) and H1-KO (F1) respectively). (C) H1-KO (F1) ESCs failed to give rise to glutamatergic neurons as compared to CTL ESCc. (D) H1-KO ESCs generated significantly lower number of CD44+ astrocytic precursors and GFAP+ astrocytes as compared to CTL ESCs (n = 1039, 0 for CTL (ATCC) and H1-KO (F1) ESCs, respectively). (E) The elaboration of dorsally derived oligodendrocyte progenitors was low in both cell lines (n = 3300, 3250). All error bars represent ±95% CI, * p<0.05 unless otherwise noted. Scale bar = 100 µm.
(TIF)

Figure S7 **Selective developmental deregulation of H1-KO (F1) ESC-derived ventral brain-specific neural lineage elaboration using the Neurobasal paradigm.** (A) There was a modest reduction in the percentage of GABAergic neurons derived from in H1-KO cell lines as compared to the CTL cell lines (n = 2723, 1037 for CTL (ATCC) and H1-KO (F1) respectively). (B) H1-KO (F1) cell lines did not give rise to TH+ dopaminergic neurons as compared to CTL cell lines. (C) The elaboration of ventrally-derived oligodendrocyte progenitors was low in both cell lines (n = 785, 800 for CTL (ATCC) and H1-KO (F1) respectively). All error bars represent ±95% CI, * p<0.05 unless otherwise noted. Scale bar = 100 µm.
(TIF)

Figure S8 **Comparative developmental gene expression profiles associated with cardiomyocyte and hepatic differentiation of CTL (ATCC) and H1-KO (F1) ESCs.** (A) *Cardiomyocyte differentiation*. CTL cell lines displayed normal acquisition of cardiomyocyte marker genes FLK-1, MHC-α, MHC-β, and MLC-2V, whereas the expression of these genes was significantly developmentally deregulated in the H1-KO (F1) cell lines. (B) *Hepatic differentiation*. The developmental profiles of gene expression of TTR, AFP, TAT and G6P were similar between CTL and H1-KO cell lines.
(TIF)

Figure S9 **Ectopic neurogenesis associated with pancreatic differentiation paradigms in H1-KO (F1) ESCs.** (A) Gene expression analysis of pancreatic genes in early pancreatic progenitors and late mature pancreatic cells in H1-KO (F1) cell lines. (B) At 33DIV, H1-KO (F1) cell lines ectopically generated β-TubIII+ neurons. (C) Gene expression analysis of proneural genes in early pancreatic progenitors in H1-KO (F1) cell lines as compared to CTL (ATCC) cell lines. All error bars represent ±95% CI; *p<0.0001 unless otherwise noted. Scale bar = 20 µm.
(TIF)

Figure S10 **Ectopic cardiomyogenesis associated with pancreatic differentiation paradigms in H1-KO (F1) ESCs.** Representative images of immunofluorescence analysis of the expression of myosin heavy chain (MF-20) present selectively in H1-KO (F1) cell line as compared to the CTL (ATCC, F18) and H1-KO (F6) cell lines. Scale bar = 20 µm.
(TIF)

Flow Chart S1 **Tissue specific differentiation paradigms.**
(TIF)

Flow Chart S2 **Experimental approach for generating neural lineage species (ITFS paradigm).**
(TIF)

Flow Chart S3 **Experimental approach for generating neural lineage species (Neurobasal paradigm).**
(TIF)

Table S1 **Summary of data obtained from CTL (ATCC) and H1-KO (F1) lines.**
(XLSX)

Table S2 **List of TaqMan probes and SYBR Green primers utilized in this study.** All TaqMan probes are listed with catalogue numbers from Applied Biosystems. All SYBR Green primers are listed with forward and reverse sequences.
(DOCX)
