# Supplementary figures and images for: The Role of H1 Linker Histone Subtypes in Preserving the Fidelity of Elaboration of Mesendodermal and Neuroectodermal Lineages during Embryonic Development
Source: PLoS One. 2014 May 6;9(5):e96858. doi: 10.1371/journal.pone.0096858 (PMC4011883; doi:10.1371/journal.pone.0096858)

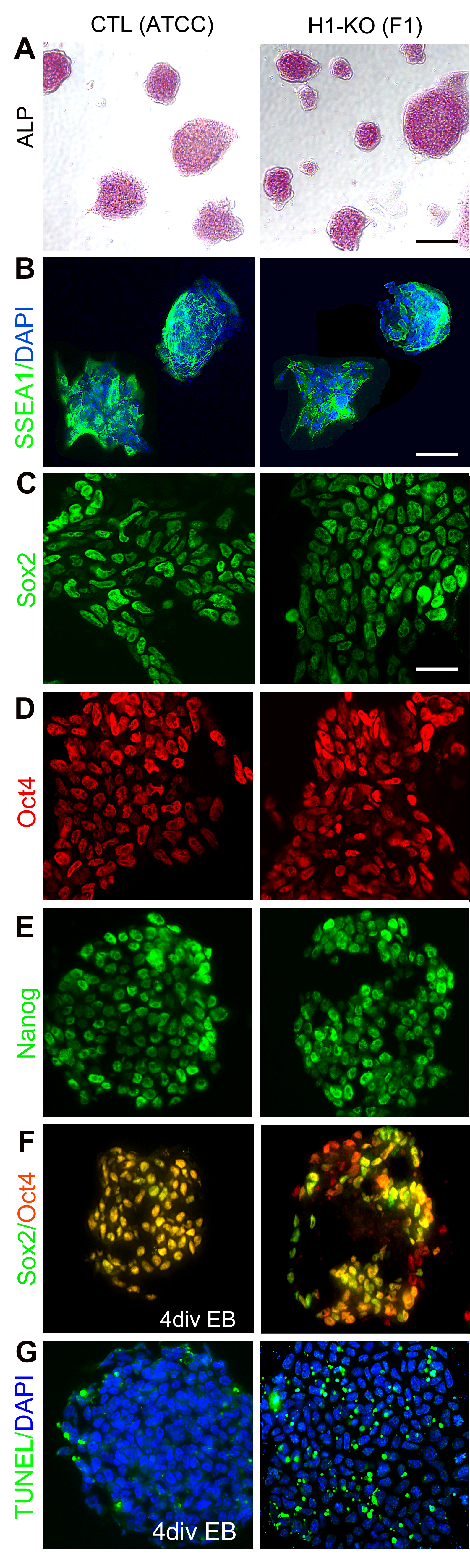

Supplement: Figure S1 — H1-KO (F1) ESCs maintain embryonic stem cell and pluripotency markers while exhibiting increased numbers of TUNEL+ cells during EB formation. (A–G) CTL (ATCC) and H1-KO (F1) ESC lines maintained expression of the embryonic stem cell markers, ALP and SSEA1, as well as the pluripotency markers, Sox2, Oct4 and Nanog (A–B, C–E). At 4 DIV, H1-KO EBs exhibited statistically significant reductions in Sox2 and Oct4 (n = 2000, 2030 for CTL (ATCC) and H1KO(F1), respectively with a p<0.001) (F) and an increase in the number of TUNEL+ cells (n = 4140, 4730 for CTL (ATCC) and H1-KO (F1), respectively, p<0.0001) (G). Scale bar = (A) 500 µm; (B) 200 µm and (C–G) 20 µm. (TIF) [file pone.0096858.s001.tif]

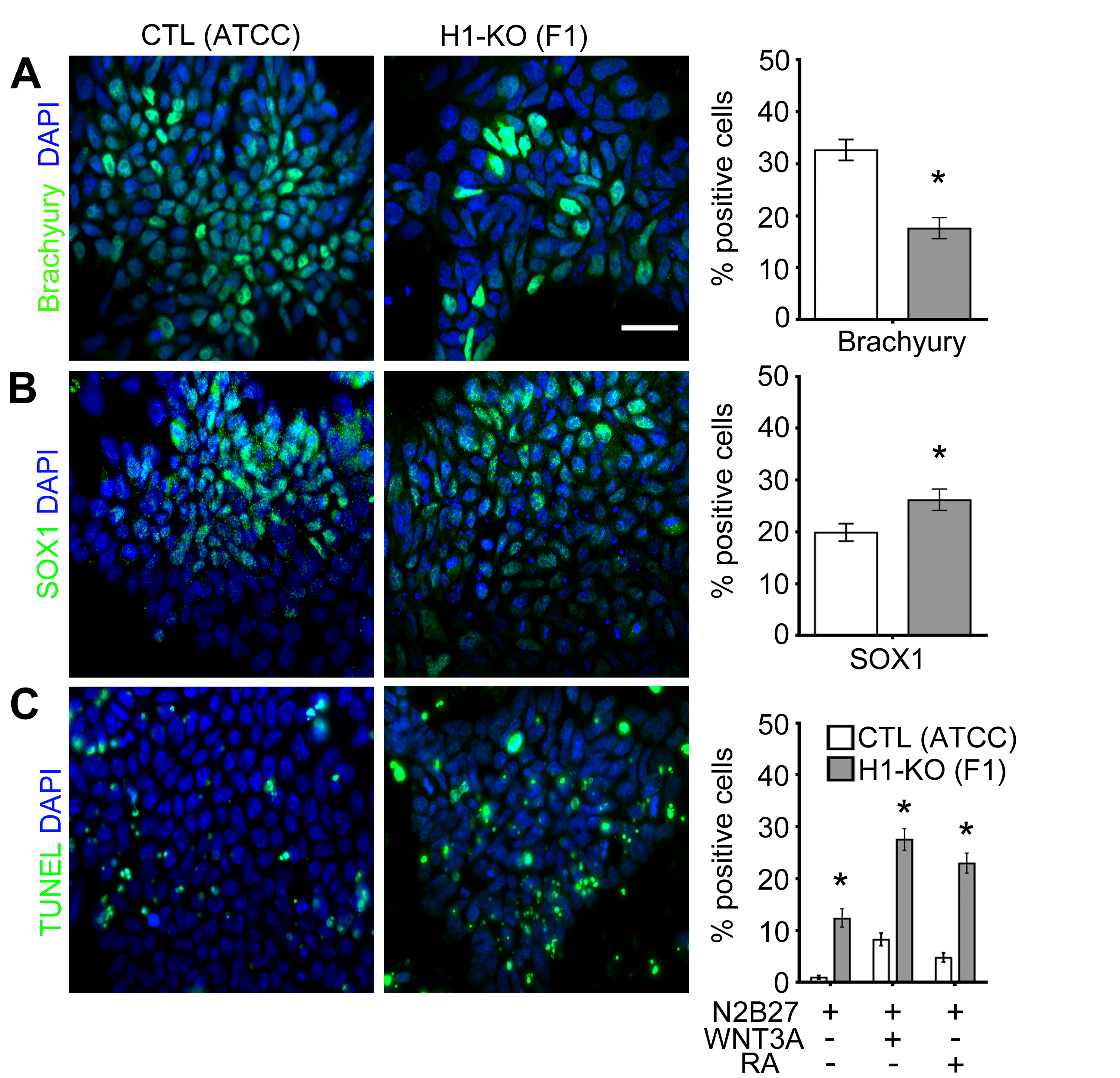

Supplement: Figure S2 — Deregulation of the induction and survival of mesendodermal and neuroectodermal cell types in H1-KO (F1) ESCs. (A) Representative images of immunofluorescence analysis of Brachyury+ mesendodermal progenitors after Wnt3A induction and the corresponding quantification of reduction in Brachyury+ cells in H1-KO (F1) cells (n = 2065, 1359 for CTL (ATCC) and H1-KO (F1), respectively). (B) Representative images of immunofluorescence analysis of SOX1+ neuroectodermal progenitors after RA induction and the corresponding quantification of SOX1+ cells in H1-KO (F1) cells (n = 2149, 1713 for CTL (ATCC) and H1-KO (F1) respectively). (C) Representative images of immunofluorescence analysis of TUNEL+ cells and quantification of TUNEL+ in different conditions (Pre-induction: n = 3000, 1315; RA-induction: 2149, 1796; Wnt3A-induction: n = 2065, 1740; for CTL (ATCC) and H1-KO (F1) respectively). All error bars represent ±95% CI; *p<0.0001 unless otherwise noted. Scale bar = 20 µm. (TIF) [file pone.0096858.s002.tif]

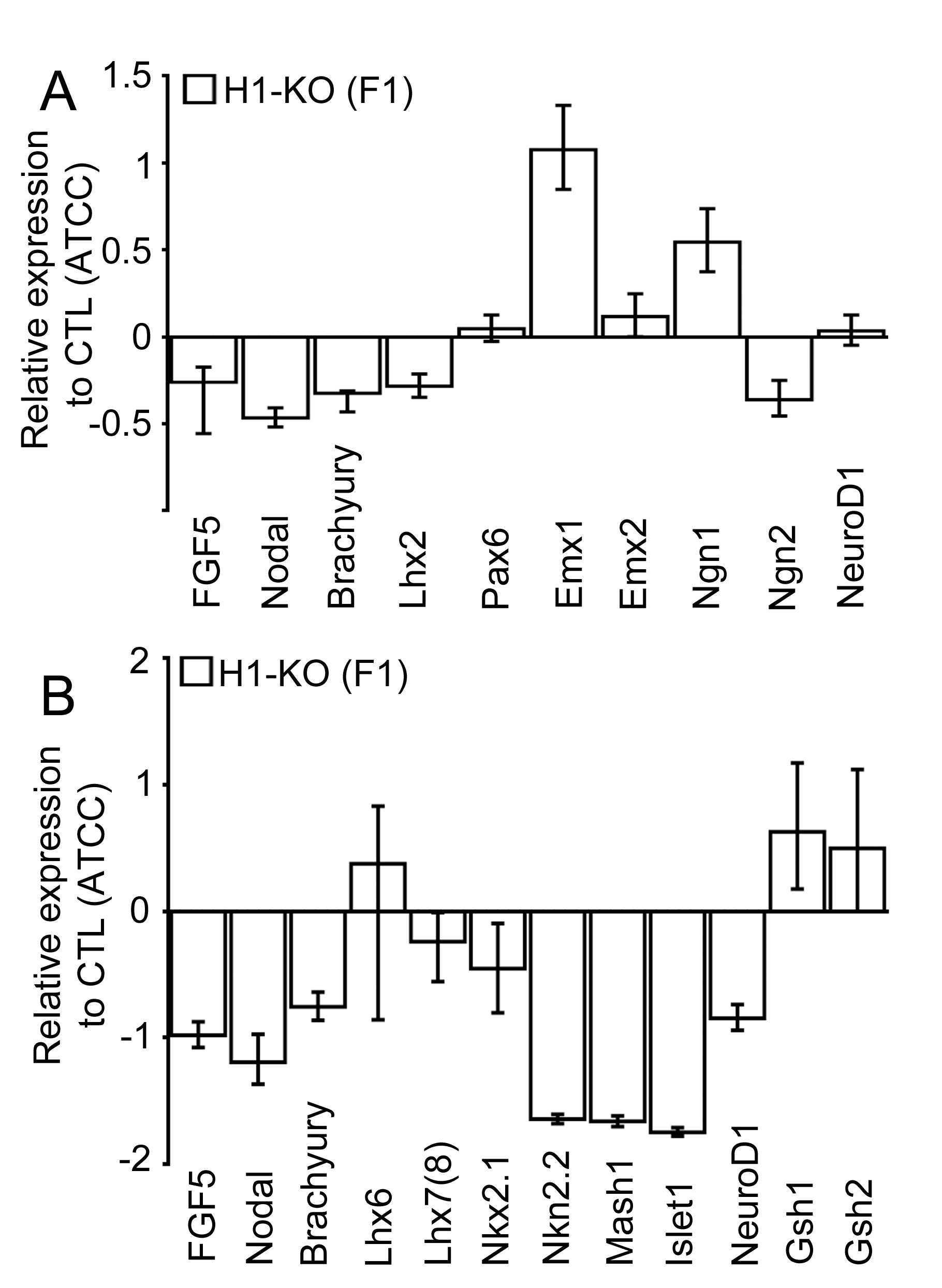

Supplement: Figure S3 — Comparative expression profiles of developmental genes in dorsal and ventral neural progenitor cell types derived from CTL (ATCC) and H1-KO (F1) ESCs using the ITFS/CM paradigm. (A) QPCR expression analysis of stage-specific developmental genes in dorsal neural progenitor cell types at 7 DIV. (B) QPCR expression analysis of stage-specific developmental genes in ventral neural progenitor cell types at 7 DIV. All error bars represent ±95% CI. (TIF) [file pone.0096858.s003.tif]

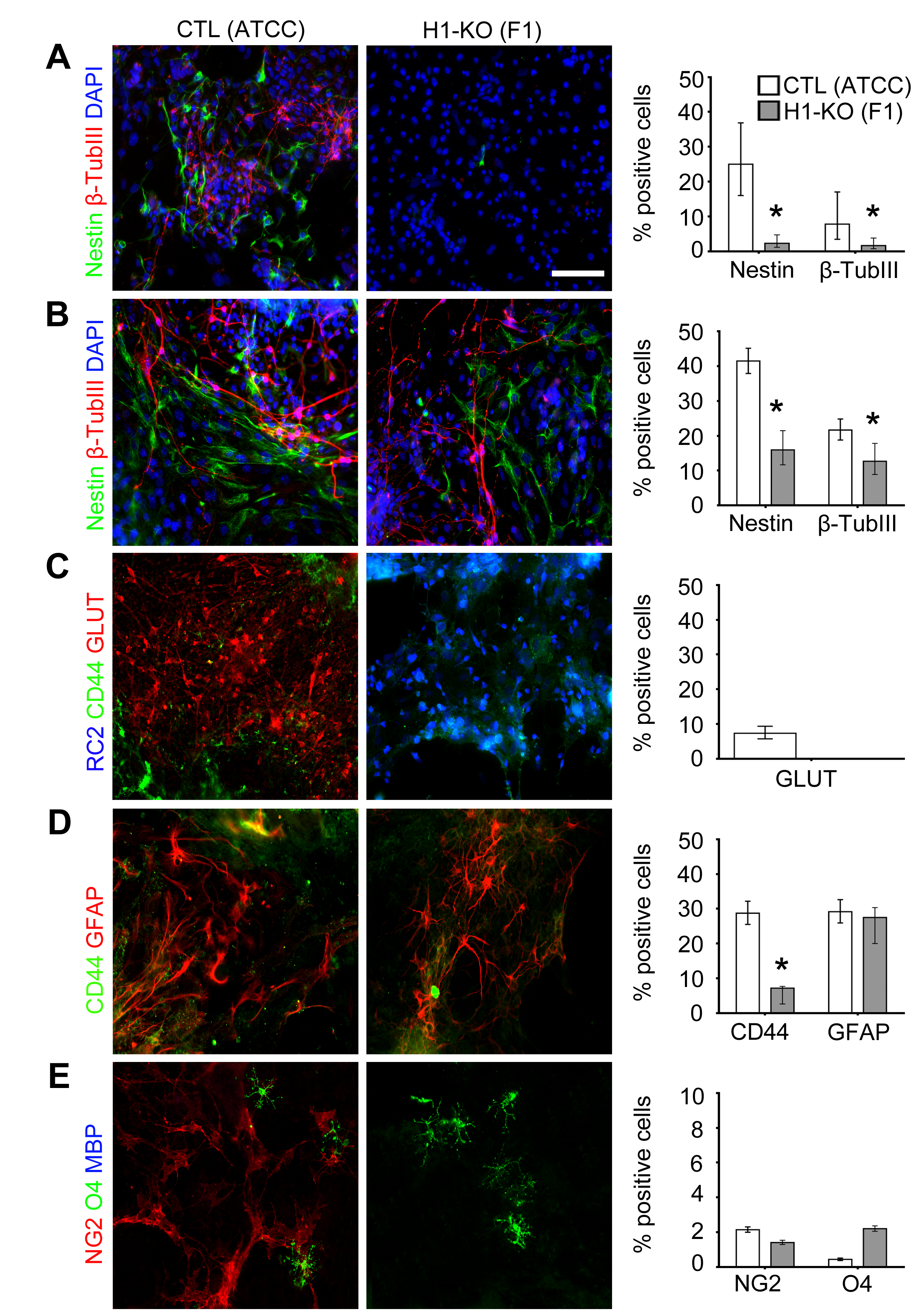

Supplement: Figure S4 — Selective developmental deregulation of H1-KO (F1) ESC-derived dorsal forebrain-specific neural lineage elaboration using the ITFS/CM paradigm. (A) CTL cell lines displayed an increased number of nestin+ NSCs and β-tubulin III+ neuronal progenitor species compared to the H1-KO cell lines (n = 64, 300 for CTL (ATCC) and H1-KO (F1) respectively). (B) Following additional in vitro maturation, CTL (ATCC) cell lines exhibited higher numbers of NSCs and neuronal progenitor species than H1-KO (F1) cell lines (n = 962, 813 for CTL (ATCC) and H1-KO (F1) respectively). (C) CTL (ATCC) cell lines showed a robust complement of glutamatergic neurons while H1-KO (F1) cell lines displayed an absence of normal neuronal differentiation. (D) CTL (ATCC) and H1-KO (F1) cell lines showed similar profiles of GFAP+ astrocytes (n = 703, 611 for CTL (ATCC) and H1-KO (F1) respectively), but H1-KO (F1) gave rise to significantly decreased number of CD44+ progenitors (n = 673, 58 for CTL (ATCC) and H1-KO. (E) The elaboration of O4+ oligodendrocyte progenitors in H1-KO (F1) cell lines was accelerated compared to those of CTL (ATCC) cell lines which predominantly has NG2+ oligodendrocyte precursors (n = 400, 1390 for CTL (ATCC) and H1-KO F1) respectively). All error bars represent ±95% CI, * p<0.05 unless otherwise noted. Scale bar = 100 µm. (TIF) [file pone.0096858.s004.tif]

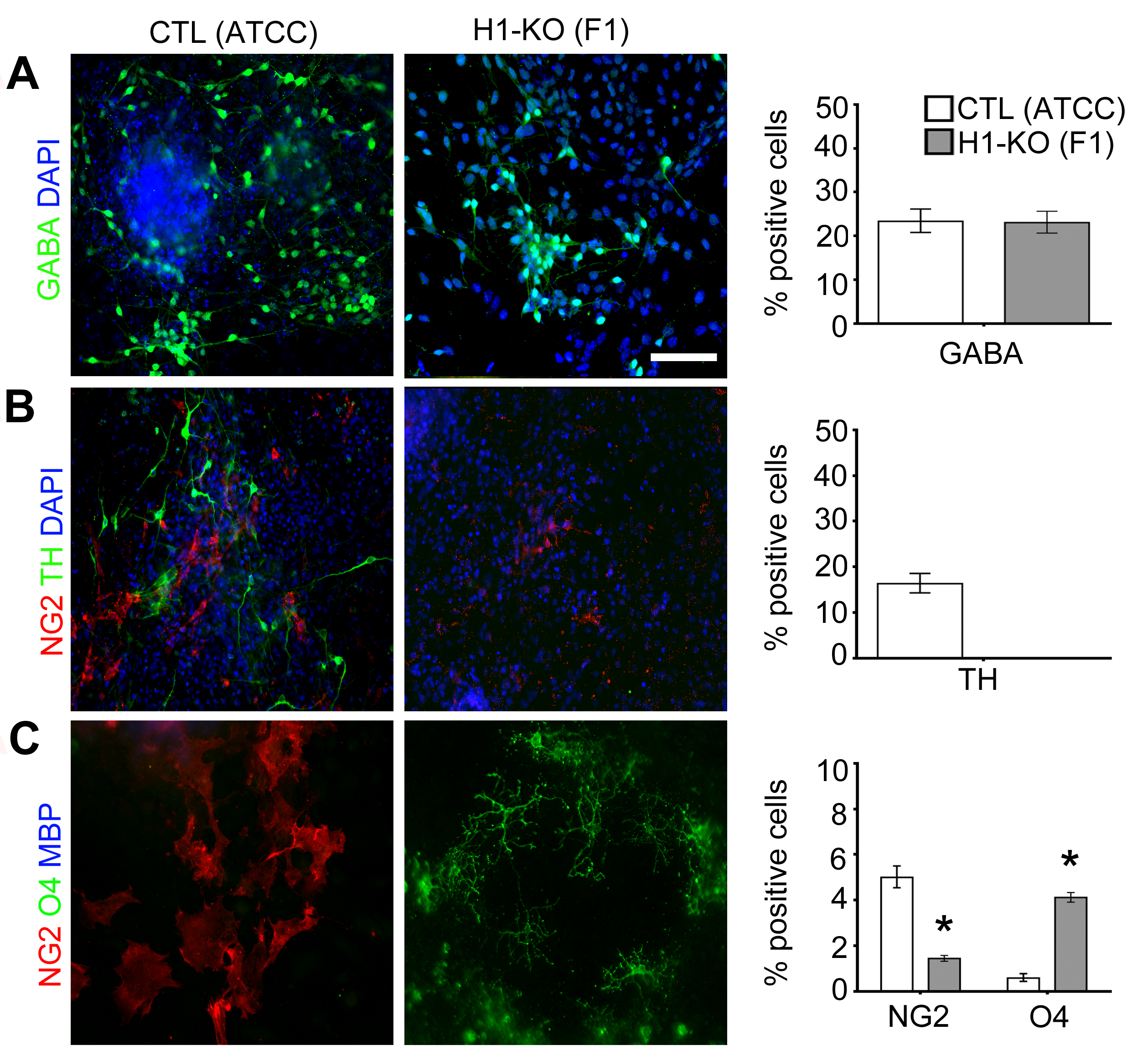

Supplement: Figure S5 — Selective developmental deregulation of H1-KO (F1) ESC-derived ventral brain-specific neural lineage elaboration using the ITFS/CM paradigm. (A) Both CTL (ATCC) and H1-KO (F1) cell lines exhibited comparable profiles of GABA+ GABAergic neurons (n = 1184 1257 for CTL (ATCC) and H1-KO (F1) respectively). (B) H1-KO (F1) cell lines do not give rise to TH+ dopaminergic neurons as compared to CTL cell lines. (C) The elaboration of O4+ oligodendrocyte progenitors in H1-KO (F1) cell lines was accelerated compared to those of CTL cell lines (n = 800, 3400 for CTL (ATCC) and H1-KO (F1) respectively). All error bars represent ±95% CI, * p<0.05 unless otherwise noted. Scale bar = 100 µm. (TIF) [file pone.0096858.s005.tif]

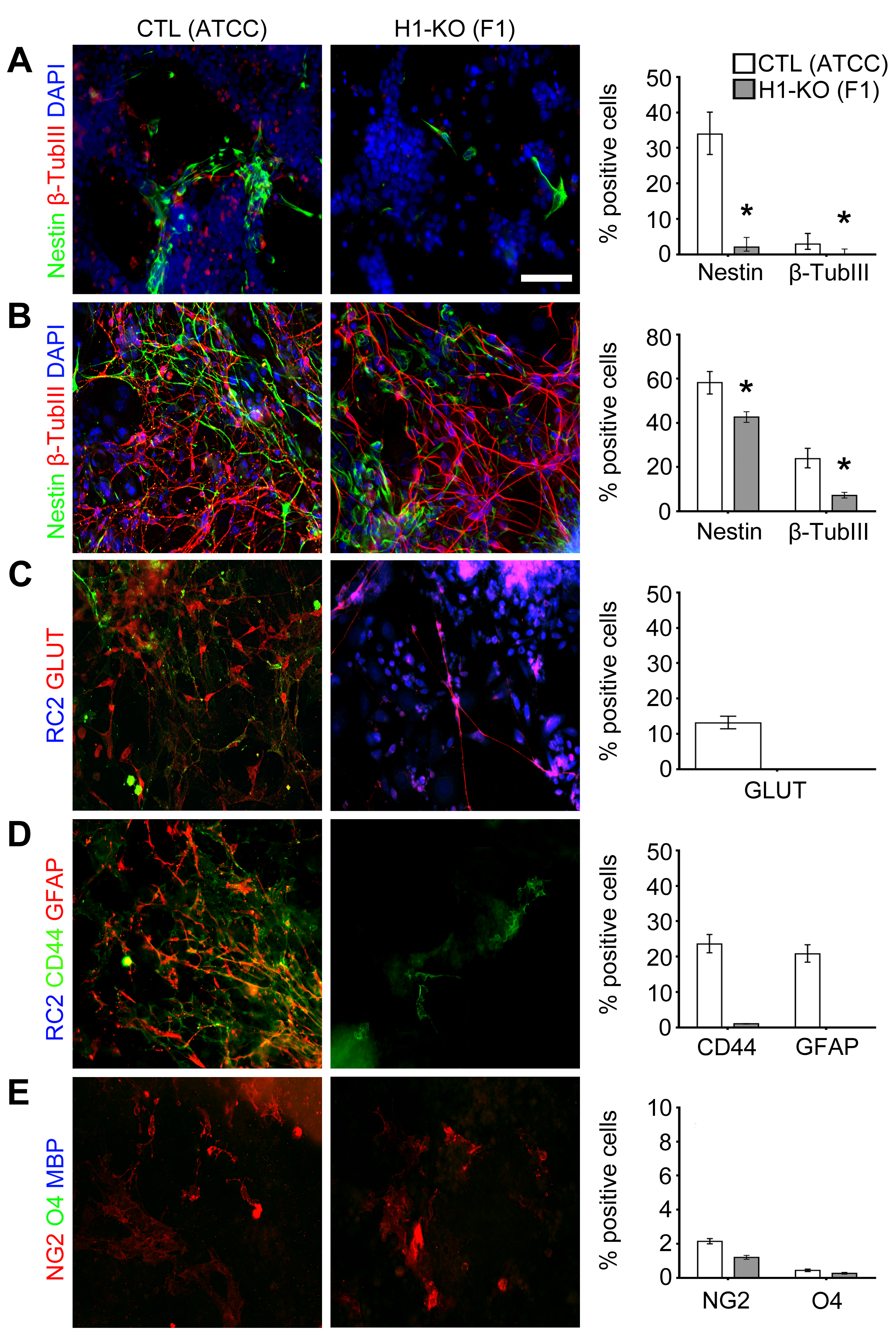

Supplement: Figure S6 — Selective developmental deregulation of H1-KO (F1) ESC-derived dorsal forebrain-specific neural lineage elaboration using the Neurobasal paradigm. (A) H1-KO (F1) cell lines generated significantly less nestin+ NSCs and β-tubulin III+ neuronal progenitor species as compared to CTL cell lines (n = 239, 238 for CTL (ATCC) and H1-KO (F1) respectively). (B) Following additional in vitro maturation, CTL cell lines generated higher numbers of nestin+ NSCs and β-tubulin III+ neuronal progenitor species than H1-KO cell lines (n = 357, 910 for CTL (ATCC) and H1-KO (F1) respectively). (C) H1-KO (F1) ESCs failed to give rise to glutamatergic neurons as compared to CTL ESCc. (D) H1-KO ESCs generated significantly lower number of CD44+ astrocytic precursors and GFAP+ astrocytes as compared to CTL ESCs (n = 1039, 0 for CTL (ATCC) and H1-KO (F1) ESCs, respectively). (E) The elaboration of dorsally derived oligodendrocyte progenitors was low in both cell lines (n = 3300, 3250). All error bars represent ±95% CI, * p<0.05 unless otherwise noted. Scale bar = 100 µm. (TIF) [file pone.0096858.s006.tif]

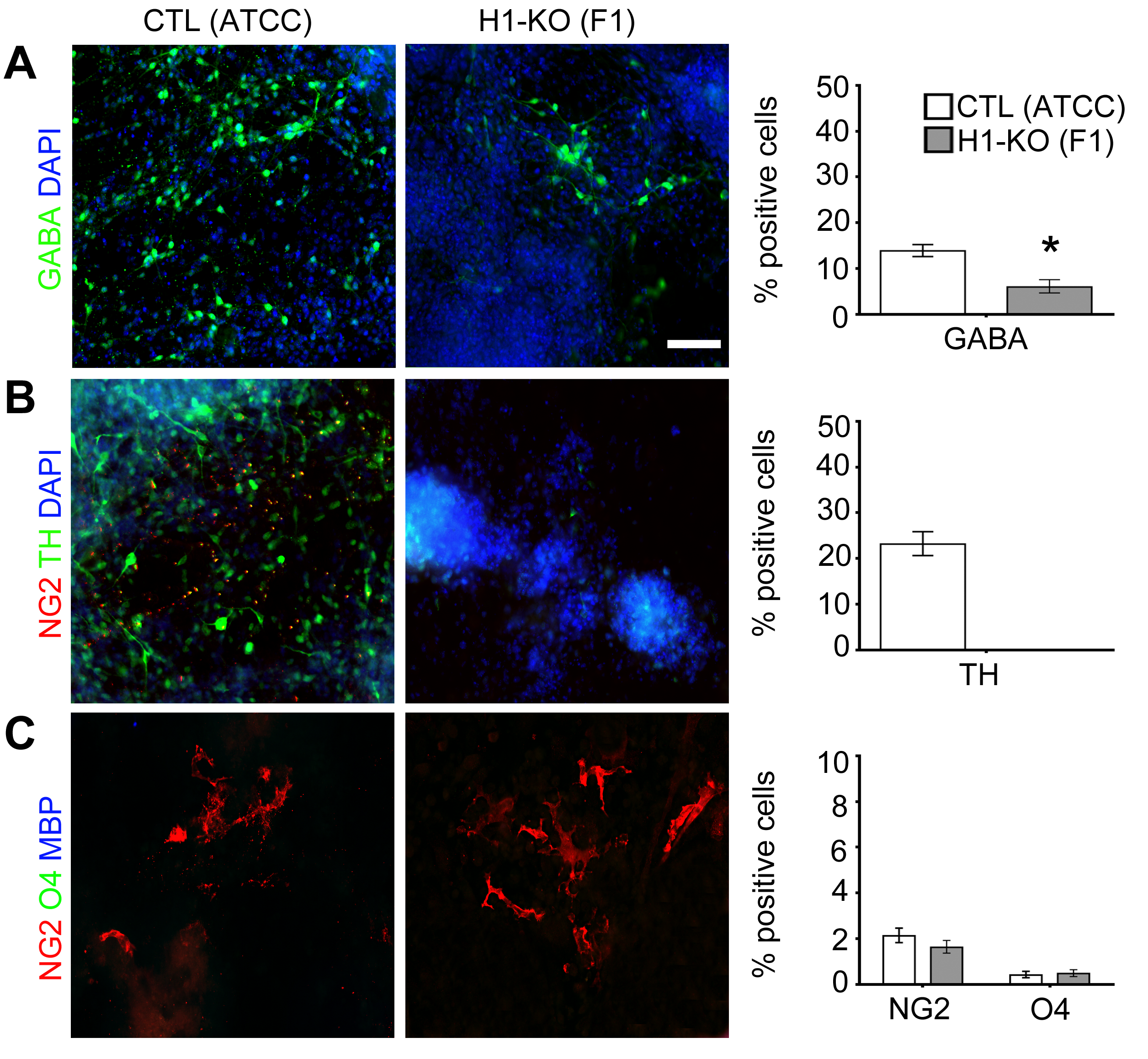

Supplement: Figure S7 — Selective developmental deregulation of H1-KO (F1) ESC-derived ventral brain-specific neural lineage elaboration using the Neurobasal paradigm. (A) There was a modest reduction in the percentage of GABAergic neurons derived from in H1-KO cell lines as compared to the CTL cell lines (n = 2723, 1037 for CTL (ATCC) and H1-KO (F1) respectively). (B) H1-KO (F1) cell lines did not give rise to TH+ dopaminergic neurons as compared to CTL cell lines. (C) The elaboration of ventrally-derived oligodendrocyte progenitors was low in both cell lines (n = 785, 800 for CTL (ATCC) and H1-KO (F1) respectively). All error bars represent ±95% CI, * p<0.05 unless otherwise noted. Scale bar = 100 µm. (TIF) [file pone.0096858.s007.tif]

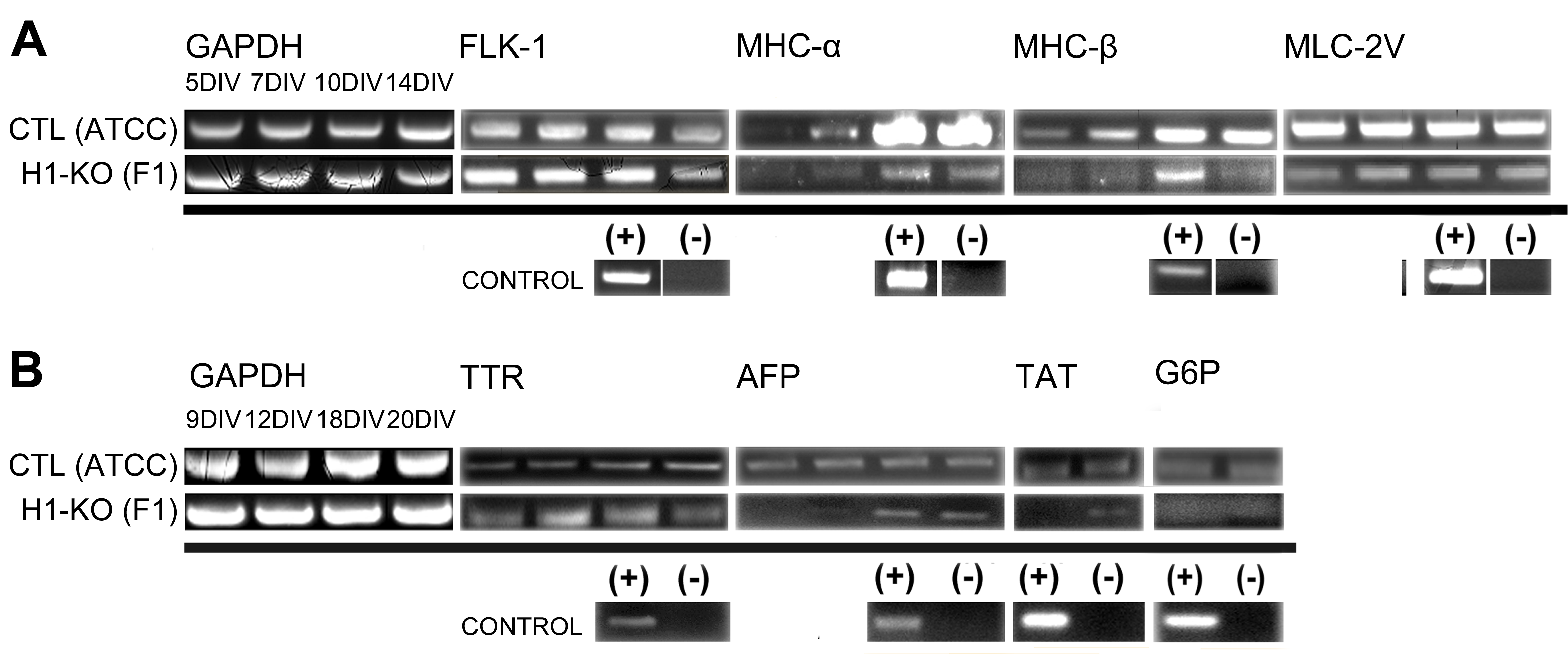

Supplement: Figure S8 — Comparative developmental gene expression profiles associated with cardiomyocyte and hepatic differentiation of CTL (ATCC) and H1-KO (F1) ESCs. (A) Cardiomyocyte differentiation. CTL cell lines displayed normal acquisition of cardiomyocyte marker genes FLK-1, MHC-α, MHC-β, and MLC-2V, whereas the expression of these genes was significantly developmentally deregulated in the H1-KO (F1) cell lines. (B) Hepatic differentiation. The developmental profiles of gene expression of TTR, AFP, TAT and G6P were similar between CTL and H1-KO cell lines. (TIF) [file pone.0096858.s008.tif]

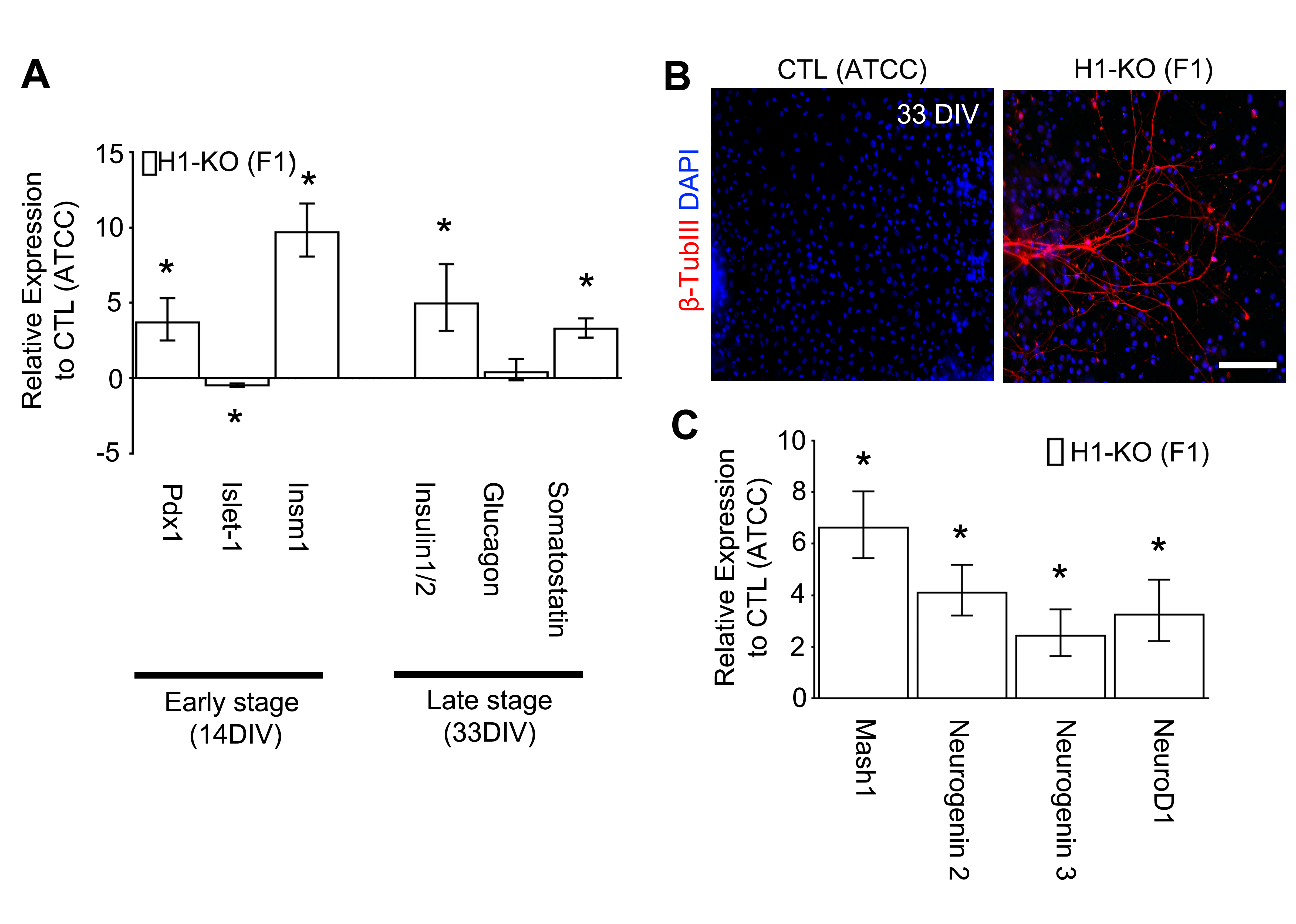

Supplement: Figure S9 — Ectopic neurogenesis associated with pancreatic differentiation paradigms in H1-KO (F1) ESCs. (A) Gene expression analysis of pancreatic genes in early pancreatic progenitors and late mature pancreatic cells in H1-KO (F1) cell lines. (B) At 33DIV, H1-KO (F1) cell lines ectopically generated β-TubIII+ neurons. (C) Gene expression analysis of proneural genes in early pancreatic progenitors in H1-KO (F1) cell lines as compared to CTL (ATCC) cell lines. All error bars represent ±95% CI; *p<0.0001 unless otherwise noted. Scale bar = 20 µm. (TIF) [file pone.0096858.s009.tif]

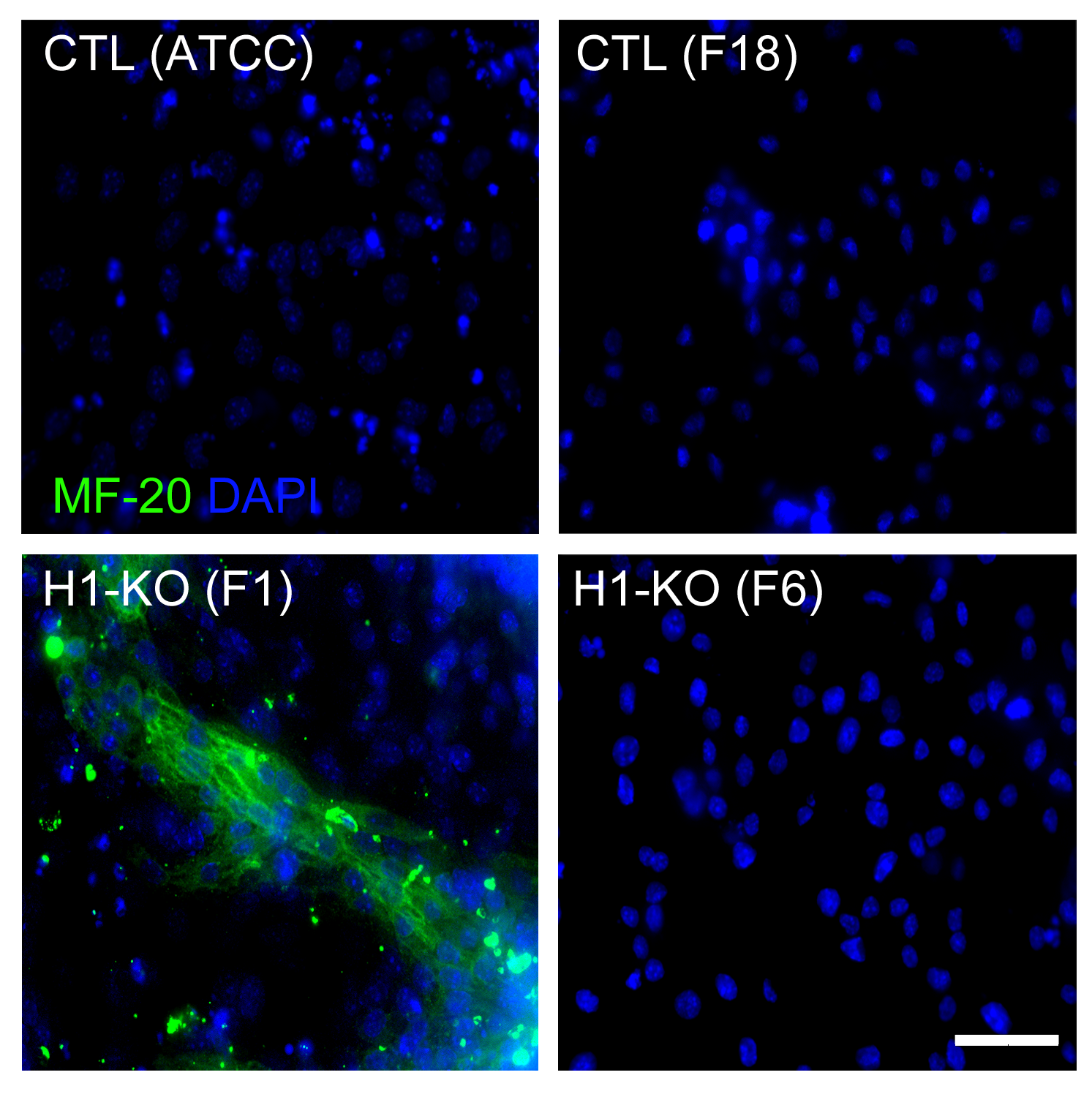

Supplement: Figure S10 — Ectopic cardiomyogenesis associated with pancreatic differentiation paradigms in H1-KO (F1) ESCs. Representative images of immunofluorescence analysis of the expression of myosin heavy chain (MF-20) present selectively in H1-KO (F1) cell line as compared to the CTL (ATCC, F18) and H1-KO (F6) cell lines. Scale bar = 20 µm. (TIF) [file pone.0096858.s010.tif]

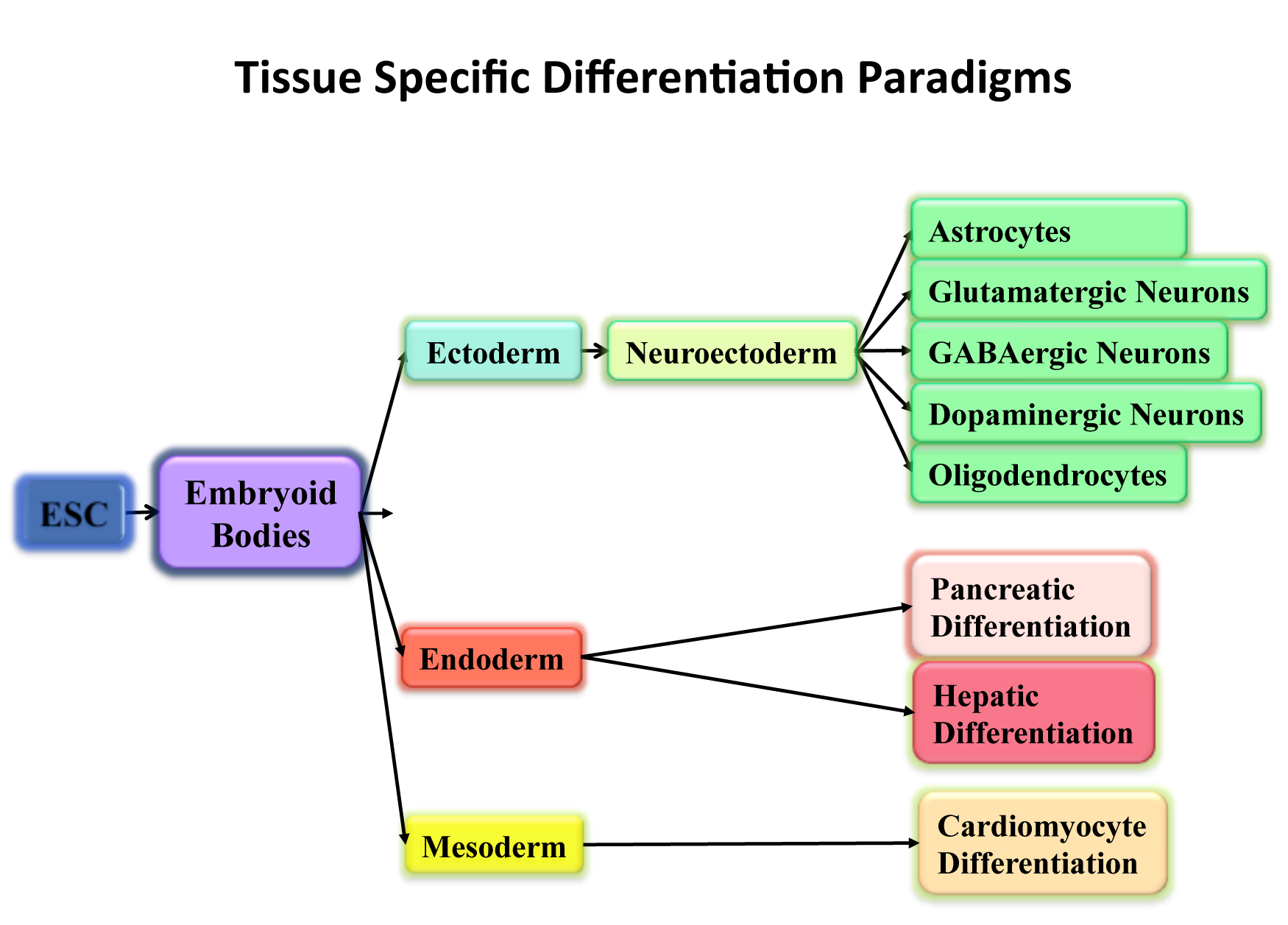

Supplement: Flow Chart S1 — Tissue specific differentiation paradigms. (TIF) [file pone.0096858.s011.tif]

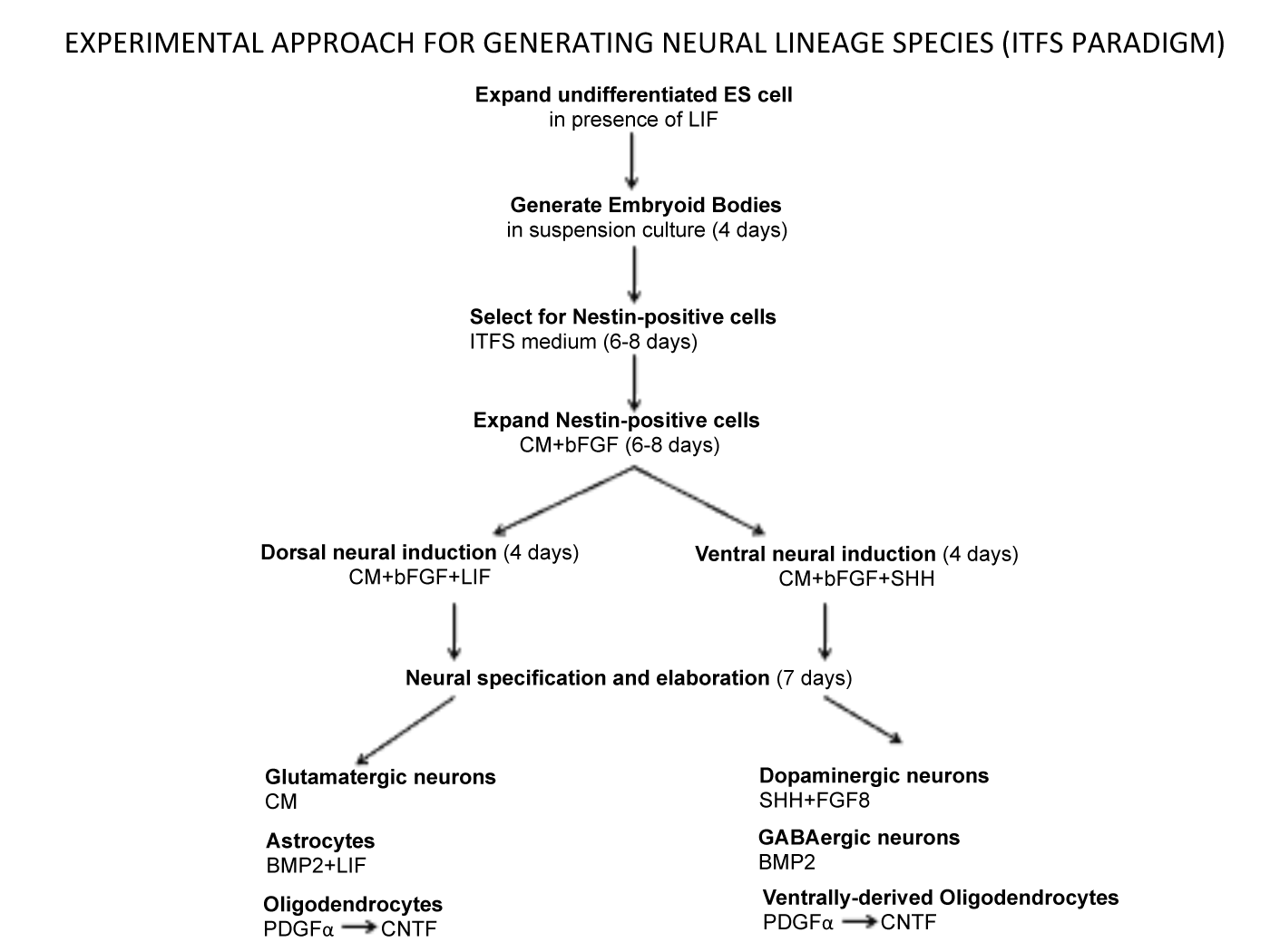

Supplement: Flow Chart S2 — Experimental approach for generating neural lineage species (ITFS paradigm). (TIF) [file pone.0096858.s012.tif]

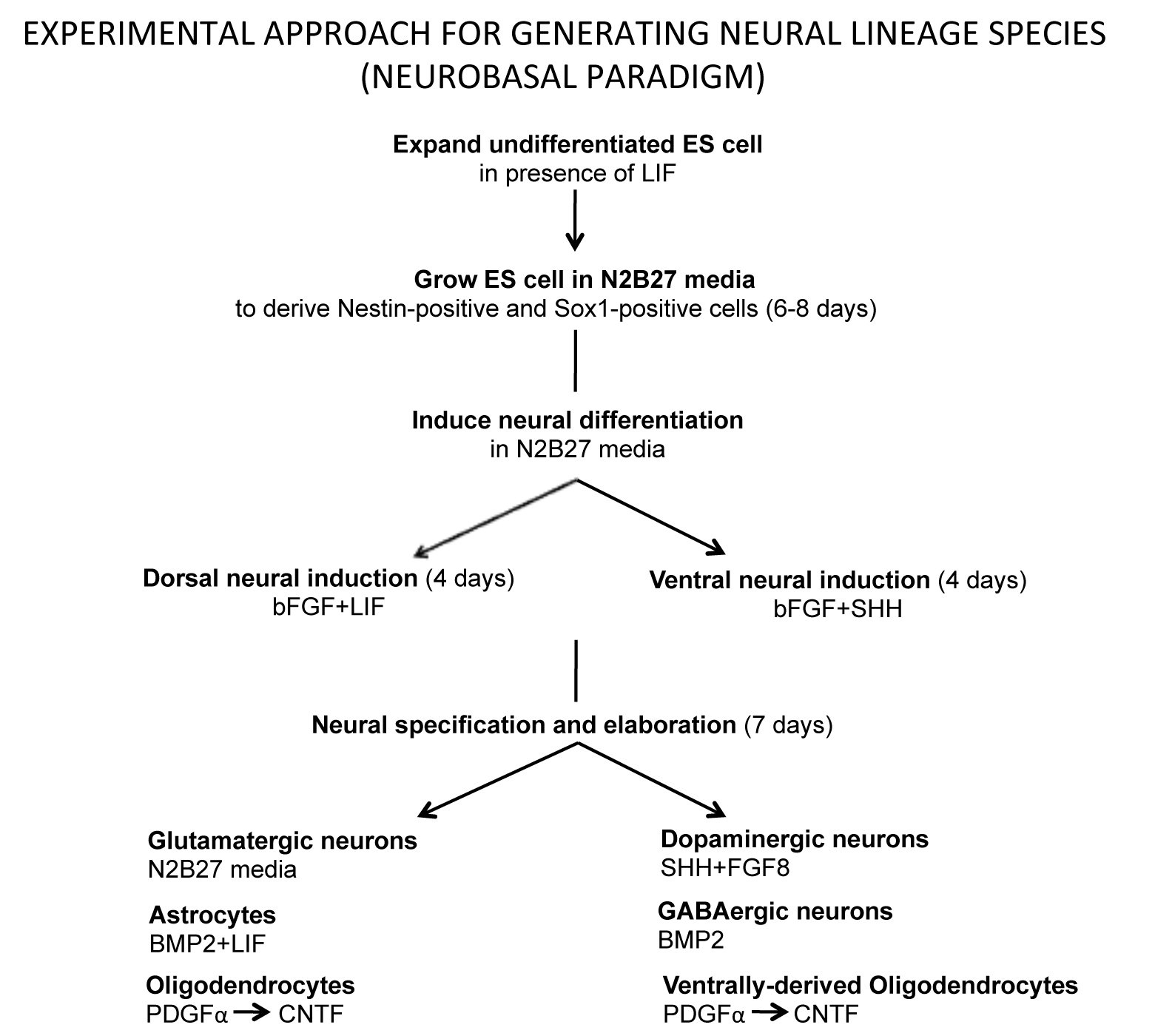

Supplement: Flow Chart S3 — Experimental approach for generating neural lineage species (Neurobasal paradigm). (TIF) [file pone.0096858.s013.tif]
