# Supplementary material for: The Role of H1 Linker Histone Subtypes in Preserving the Fidelity of Elaboration of Mesendodermal and Neuroectodermal Lineages during Embryonic Development
Source: PLoS One. 2014 May 6;9(5):e96858. doi: 10.1371/journal.pone.0096858 (PMC4011883; doi:10.1371/journal.pone.0096858)
Supplement: Table S2 — List of TaqMan probes and SYBR Green primers utilized in this study. All TaqMan probes are listed with catalogue numbers from Applied Biosystems. All SYBR Green primers are listed with forward and reverse sequences. (DOCX) [file pone.0096858.s015.docx]

**Table S2**

| **TaqMan Probes** | | **Applied Biosystems catalogue number** |
| --- | --- | --- |
| HPRT1 | | Mm00446968_m1 |
| FGF5 | | [Mm00438918_m1](https://products.appliedbiosystems.com:443/ab/en/US/adirect/ab?cmd=ABAssayDetailDisplay&assayID=Mm00438918_m1&Fs=y&adv_phrase3=EXACT&adv_phrase2=EXACT&adv_phrase1=EXACT&assayType=GE&catID=601267&batchSearchDecider=textarea&adv_kw_filter3=ALL&srchType=keyword&adv_kw_filter2=ALL&SearchRequest.Common.QueryText=14176&adv_kw_filter1=ALL&adv_query_text3=&searchType=keyword&adv_query_text2=&adv_query_text1=&uploadType=ID+List&adv_boolean3=AND&adv_boolean2=AND&adv_boolean1=AND&chkBatchQueryText=false&kwfilter=ENTREZGENE_ID&SearchRequest.Common.PageNumber=1&msgType=ABGEKeywordResults) |
| NODAL | | [Mm00443040_m1](https://products.appliedbiosystems.com:443/ab/en/US/adirect/ab?cmd=ABAssayDetailDisplay&assayID=Mm00443040_m1&Fs=y&adv_phrase3=EXACT&adv_phrase2=EXACT&adv_phrase1=EXACT&assayType=GE&catID=601267&batchSearchDecider=textarea&adv_kw_filter3=ALL&srchType=keyword&adv_kw_filter2=ALL&SearchRequest.Common.QueryText=18119&adv_kw_filter1=ALL&adv_query_text3=&searchType=keyword&adv_query_text2=&adv_query_text1=&uploadType=ID+List&adv_boolean3=AND&adv_boolean2=AND&adv_boolean1=AND&chkBatchQueryText=false&kwfilter=ENTREZGENE_ID&SearchRequest.Common.PageNumber=1&msgType=ABGEKeywordResults) |
| BRACHYURY | | [Mm01318252_m1](https://products.appliedbiosystems.com:443/ab/en/US/adirect/ab?cmd=ABAssayDetailDisplay&assayID=Mm01318252_m1&Fs=y&adv_phrase3=EXACT&adv_phrase2=EXACT&adv_phrase1=EXACT&assayType=GE&catID=601267&batchSearchDecider=textarea&adv_kw_filter3=ALL&srchType=keyword&adv_kw_filter2=ALL&SearchRequest.Common.QueryText=20997&adv_kw_filter1=ALL&adv_query_text3=&searchType=keyword&adv_query_text2=&adv_query_text1=&uploadType=ID+List&adv_boolean3=AND&adv_boolean2=AND&adv_boolean1=AND&chkBatchQueryText=false&kwfilter=ENTREZGENE_ID&SearchRequest.Common.PageNumber=1&msgType=ABGEKeywordResults) |
| Pax6 | | Mm00443081_m1 |
| Ngn1 | | Mm00440466_s1 |
| Ngn2 | | Mm00437603_g1 |
| Emx2 | | Mm00550241_m1 |
| Gsh1 | | Mm04207461_g1 |
| Gsh2 | | Mm00446650_m1 |
| **SYBR Green Probes** | | **Forward/Reverse Sequences** |
| HPRT1 | | CAGTCCCAGCGTCGTGATTA / GAATAAACACTTTTTCCAAATCCTCG |
| Pancreatic Genes | Pdx1 | AAGGCCAGTGGGCAGGAGGT / TCCTTCTCCAGCTCCAGCAGCT |
|  | Hes1 | GCAGATGACCGCCGCGCTC / CGCCCTCACACGTGGACAGG |
|  | Sox9 | GCTATCTTCAAGGCGCTGCAAGC / GCGGACCCTGAGATTGCCCAGA |
|  | Insm1 | CCGAGCGCCAAAAAGCCGAA / TCCACCGGGCCCTCCTTGAT |
|  | NeuroD1 | CAATCTTCTCTTCCGGTGCCGC / CTGGGCACTCATGACTCGCTCA |
|  | Ngn3 | AGCCTCGGACCACGAAGTGC / TCCCTCGGCAGTCACCCACT |
|  | Islet1 | GCCTCTGCAAATGGCAGCCGA / ACAGTCCGCACTCGGGTGGT |
|  | Insulin1/2 | TGGAGGACCCACAAGTGGCACA / ATGCCACGCTTCTGCTGGGC |
|  | Glucagon | ACCCCAGATCATTCCCAGCTTCC / AGTCGCTGGTGAATGTGCCCT |
|  | Somatostatin | CACCGGGAAACAGGAACTGGCC / GCTCAGCTGCCTGGGGCAAA |
| Neural Genes | Lhx2 | ACACGGAGACGACCATGC / AGCAGGTAGTAGCGGTCAGA |
|  | Lhx6 | GGCCCATGTACTGGAAGCAT / CTGGGCCATCACCTGGTC |
|  | Lhx7(8) | GCTCCAGGTTATGCAAGCAC / ACGTCTGCTTAAGCCTGTCC |
|  | Nkx2.1 | GGACTGGGATGTCCTCGGAA / TGATTCGGCGTCGGCTGG |
|  | Nkx2.2 | ACCTGGCCAGCCTCATCCGT / TGAAATGCTTTCTCCGCCCGGG |
|  | Mash1 | CTACGACCCTCTTAGCCCAG / TGCCATCCTGCTTCCAAAGTC |
|  | Islet1 | GCCTCTGCAAATGGCAGCCGA / ACAGTCCGCACTCGGGTGGT |
|  | Emx1 | CTTCCAGGCAAGCGACGTT / GCGAGAAGGCTGTGCGAATC |
|  | NeuroD1 | ACCTTTTAACAACAGGAAGTGGA / CTCATCTGTCCAGCTTGGGG |
